# Nutritional management of growth faltering in infants aged under six months in Asia and Africa: study protocol for a multicentre randomised trial (BRANCH, *BR*e*A*stfeedi*N*g *C*ounselling and management of growt*H*)

**DOI:** 10.1186/s13063-025-09034-y

**Published:** 2025-11-06

**Authors:** WHO BRANCH study group, Salahuddin Ahmed, Abdullah H Baqui, Md. Monzur Rahman Bhuiyan, Rukhsana Haider, Ashraful Islam, Ahad M Khan, Rasheda Khanam, Kayur Mehta, Arunangshu D Roy, Rezwana Tabassum, Semira Abdelmenan, Hanna Y Berhane, Yemane Berhane, Nebiyou Fasil, Wafaie W Fawzi, Uttara Partap, Christopher R Sudfeld, Dongqing Wang, Alemayehu Worku, Nita Bhandari, Kiran Bhatia, Harish Chellani, Ranadip Chowdhury, Kriti Jain, Sowmya Karantha C, Jasmine Kaur, Sitanshi Sharma, Girish Chand Pant, Sunita Taneja, Ebunoluwa A. Adejuyigbe, Henry C. Anyabolu, Ibrahim O. Awowole, Olabisi Iyabode Dedeke, Bankole P. Kuti, Tolulope Ogundele, Aysha Arif, Amina Barkat, Saleema Gulzar, Syed Muhammad Umair Hamid, Fyezah Jehan, Zohra Kurji, Amal Fatima Mohiuddin, Usma Mehmood, Junaid Mehmood, Muhammad Imran Nisar, Salman Osmani, Salma Rattani, Bishara Ali Alawi, Siti Makame Ali, Farhad Athad, Jamila Khalfan Ali, Sayan Das, Dr Saikat Deb, Usha Dhingra, Arup Dutta, Khamis Salim Mussa, Maryam Fihri Said, Sunil Sazawal, Amanda Murungi Eunice, Dan Kajungu, Sarah Kiguli, Grace Ndeezi, Ezekiel Mupere, Philippa Musoke, Nicollette Nabukeera, Jesca Nsungwa, James Tumwine, Peter Waiswa, Rajiv Bahl, Rhian Daniel, Karen M Edmond, Ameena Goga, Lisa Hurt, Natalie Strobel, Nigel Rollins

**Affiliations:** Organisation mondiale de la Sante, Geneva, Switzerland

**Keywords:** Growth, Infant, Malnutrition, Breastfeeding

## Abstract

**Background:**

Treatment of growth faltering in early infancy may improve short and long term child health outcomes. The overall aim of this trial is to determine, in infants who meet study criteria for growth faltering, the effect of intensive breastfeeding counselling and support (IBFCS) plus nutritional milk supplementation (NMS) compared with IBFCS alone, on mortality, morbidity and growth at 6 completed months in low resource settings in South Asia and Sub-Saharan Africa. The primary outcome of the trial is wasting free survival (alive without wasting (weight for length standard deviation score < − 2 standard deviations (SD))) at 6 completed months of age.

**Methods:**

This is a multi-centre, parallel-group, individually-randomized, non-blinded, controlled trial implemented in seven countries: three in Asia (Bangladesh, India and Pakistan) and four in Africa (Ethiopia, Nigeria, Tanzania and Uganda).

Eleven thousand (11,000) infants with a gestational age of at least 28 weeks are enrolled and individually randomised between 7 and 14 days of age. The mother of each infant receives breastfeeding support from trained peer counsellors. Research workers follow up each infant 1–2 weekly at the infant’s home to collect growth and outcome data. If infants meet study criteria for growth problems (slow weight gain, growth concern or growth faltering) they are reviewed by study clinicians, their medical problems are treated, and their mothers receive IBFCS. Infants with growth faltering in the intervention arm also receive nutritional milk supplementation (NMS) (prescribed quantities of term infant formula that meets Codex Alimentarius standards calculated to fulfill the needs for catch up growth). The comparison group receives IBFCS alone. Weekly growth monitoring continues and final outcome data (mortality, wasting) are measured in all infants at 6 completed months.

**Discussion:**

This large randomised trial will provide evidence about the role of NMS, if any, in infants with growth faltering who do not respond to IBFCS and treatment of medical problems in low resource settings.

**Trial registration:**

Australian and New Zealand Clinical Trial Registry (ANZCTR) CTRN12624000704594. Registered on June 4 2024.

**Supplementary Information:**

The online version contains supplementary material available at 10.1186/s13063-025-09034-y.

## Introduction

Growth is an important marker of resilience in infants and young children [1]. The first 6 months of life are a critical period for health, survival, growth and neurodevelopment for both preterm and term infants [1]. Declines in growth velocity (commonly called growth faltering or ‘failure to thrive’) [2, 3], in the first 6 months of life, are associated with high risks of mortality, morbidity and poor long term neurodevelopmental outcomes [2].

The most important intervention to prevent and manage growth faltering in the first 6 months is the promotion and support of exclusive breastfeeding, including responsive feeding, intensive lactation management when required, and care for infant and maternal health and nutrition [4–6]. However, despite support for breastfeeding, some infants falter in growth in the first 6 months of life [2]. Causes include chronic infections and disease, insufficient neurodevelopmental stimulation, lack of responsive caregiving and poor maternal health and wellbeing [2].

There are clear World Health Organization (WHO) guidelines for the management of moderate and severe acute malnutrition in the first 6 months of life [2]. However, there are no WHO guidelines for early intervention and management of growth faltering over this period [2, 7]. The optimal treatment for growth faltering in infants aged under 6 months remains unknown, and no intervention studies have examined this issue. It is unclear if the provision of a nutritional milk supplement (NMS) has any benefit for an infant whose mother is already receiving intensive breastfeeding counselling and support (IBFCS). It is also uncertain if any potential benefit of NMS would outweigh the risks using NMS such as incorrect preparation of feeds (over and under dilution), use of household water supplies to prepare the NMS and other household contamination of the NMS (e.g. with soil or other household items). Evaluation of interventions to manage growth faltering in the first 6 months after birth was one of the highest priority research questions identified during an informal WHO consultation in January 2019 [7].

The WHO Newborn and Child Health and Development Unit (WHO/NBC) is coordinating a multi-country, multi-centre randomised controlled trial to assess if NMS has any effect in growth faltering infants in addition to IBFCS in low resource settings [8, 9]. The trial is being conducted in study populations with a high burden of underweight, wasting, low birth weight (LBW) and preterm birth in infants aged under 6 months in seven countries in South Asia and Sub-Saharan Africa. This paper describes the protocol developed for the trial and follows the SPIRIT (Standard Protocol Items: Recommendations for Interventional Trials 2013) format [10].

## Aims, objectives and outcomes

### Aim

The overall aim of this trial is to determine, in infants who meet study criteria for growth faltering, the effect of intensive breastfeeding counselling and support (IBFCS) plus nutritional milk supplementation (NMS) compared with IBFCS alone on mortality, morbidity and growth at 6 completed months in low resource settings in South Asia and Sub-Saharan Africa.

### Objectives

The *primary objective* of the trial is to determine the effect of NMS on wasting free survival at 6 completed months of age (alive without wasting (weight for length standard deviation score (WLZ score) < −2 standard deviations (SD)).

*Secondary objectives* of the trial are the following:

- To determine the effect of NMS on the primary outcome in subgroups based on birth weight and gestational age at birth (term appropriate for gestational age (AGA), preterm AGA, term small for gestational age (SGA), preterm SGA); and to determine the effect of NMS on the secondary outcomes as listed in Table 1. 

To determine the effect of NMS on the secondary outcomes as listed in Table 1.

**Table 1:** Primary and secondary outcome definitions, timing and method of measurement

Outcome	Definition	Time point for data collection	Method of measurement
*Primary outcome*			
Wasting free survival	Alive without wasting (weight for length z score < − 2 standard deviations (SD))	At 6 completed months	Mother’s recall of event in the previous 2 weeks (all-cause mortality) Weight and length measurements taken by trained field workers (wasting)
*Secondary outcomes*			
Mortality	All-cause mortality	At 6 completed months	Mother’s recall of event in the previous 2 weeks
Underweight	Weight-for-age z-score < − 2 SD	At 6 completed months	Measurements taken by trained field workers
Wasting	Weight for length z-score < − 2 Sd	At 6 completed months	Measurements taken by trained field workers
Severe wasting	Weight for length z-score < − 3 SD	At 6 completed months	Measurements taken by trained field workers
Stunting	Length for age z-score < − 2 Sd	At 6 completed months	Measurements taken by trained field workers
Concurrent wasting and stunting	Composite measure of wasting and/or stunting	At 6 completed months	Measurements taken by trained field workers
All-cause morbidity	Any hospitalisation (defined as either an inpatient admission (where registration number is allotted) or a stay of more than or equal to 24 consecutive hours in the treatment facility/hospital (excluding waiting time) for any cause	From enrolment until 6 completed months	Mother’s recall of event in the previous 2 weeks
Cause-specific morbidity	Any hospitalisation in the previous 2 weeks for diarrhoea, pneumonia, other infectious disease, other cause	From enrolment until 6 completed months	Mother’s recall of event in the previous 2 weeks
Breastfeeding practices	Receipt of mother’s own breastmilk in the previous 24 h	At 6 completed months	Mother’s recall of event in the previous 24 h
Careseeking practices	Any health care-seeking in the previous 2 weeks, including any face-to-face visit to any health facility, pharmacist or drug-seller, but excluding online or telephone consultations	From enrolment until 6 completed months	Mother’s recall of event in the previous 2 weeks

*SD* standard deviation

## Design and timeline

### Trial design

**Fig. 1:**
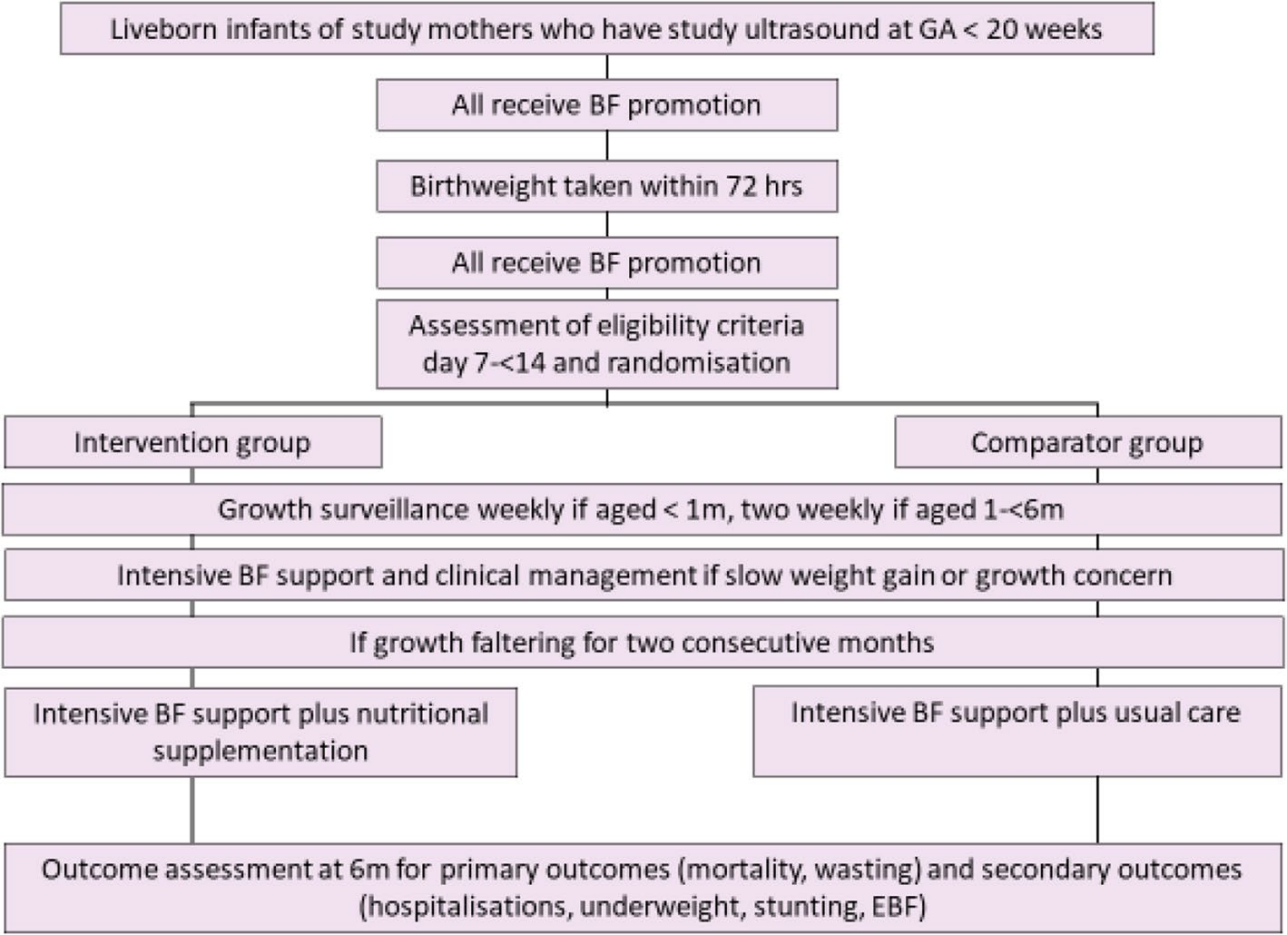
Study flow

This is a multi-centre, parallel-group, individually-randomised, non-blinded, controlled, superioritytrial implemented in seven countries: three in Asia (Bangladesh, India and Pakistan) and four in Africa (Ethiopia, Nigeria, Tanzania and Uganda). CONSORT guidelines will be followed in the reporting of the trial [11, 12]. The allocation ratio of intervention and comparison groups is 1:1, and the trial flow is shown in Fig. 1. The trial is registered with the Australian and New Zealand Clinical Trial Registry (ANZCTR) CTRN12624000704594.

### Participant timeline

**Fig. 2:**
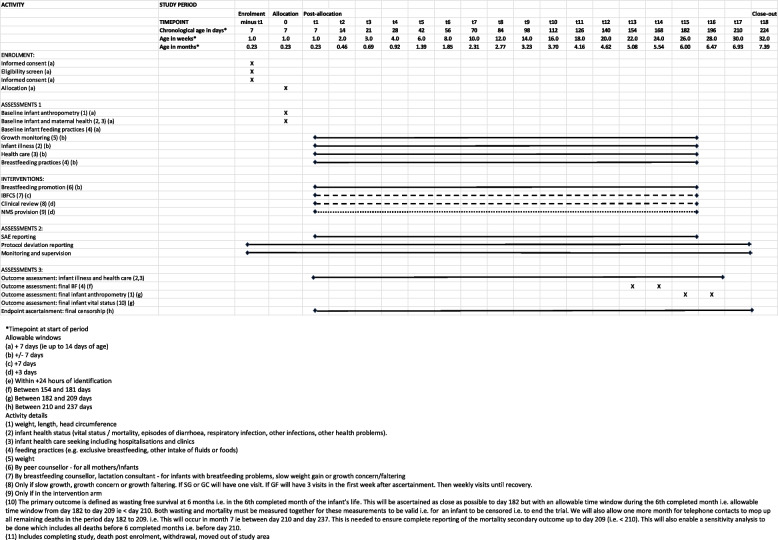
Trial activity matrix (SPIRIT matrix)

Enrolment and follow up is conducted over 24 months (infants are enrolled over 18 months and followed up until they reach 6 months). The trial activity matrix is shown in Fig. 2, and the field work organisation is shown in Appendix 1.

## Setting and organisation

### Study organisation

WHO is the sponsor of the trial and the Bill & Melinda Gates Foundation (BMGF) is the funder. There are designated principal investigators for each site. WHO leads the trial coordinating unit (TCU) which consists of WHO, the principal investigators for each site, the trial statistics team and the trial data management team. The TCU is responsible for coordination of the study and meets monthly. Each site has dedicated teams for pregnancy and infant surveillance, independent outcome assessment (IOA), breastfeeding, clinical support, intervention delivery and data management. Each team has their own coordinator who trains, supports and monitors their team. There is an external advisory group (EAG) consisting of four senior epidemiology and nutrition experts. The EAG provides expert advice throughout all stages of the trial and reviews progress each year. An independent Data Safety Monitoring Board (DSMB) was constituted prior to the start of the trial with terms of reference (TOR) to review data, monitor the progress of the trial and assess the safety of the interventions. The DSMB advise on continuation, modification or termination based on pre-decided stopping rules. The DSMB meets 3 monthly and as needed (Appendix 4).

### Study setting

The sites were chosen by the sponsor after an open call for expressions of interest. The eligbility criteria can be found in Appendix 5. Table 2 summarises the key characteristics of the study sites. The study is conducted in geographically defined urban and rural catchment populations in the seven countries. Women of reproductive age in the households in the catchment populations are consented to participate in reproductive and pregnancy surveillance. Women are asked if they think they might be pregnant or if they have missed any periods. Pregnant women are offered: a trans-abdominal ultrasound which is scheduled as early as possible and anthropometry (measurement of weight, height and mid-upper arm circumference (MUAC)). Women who are less than 20 weeks gestational age at the time of the ultrasound are followed up so that the research team can be notified of the birth and the birth weight measured by 72 h post-partum.

**Table 2:** Characteristics of study sites

	Ethiopia	Bangladesh	Pakistan	India	Nigeria	Tanzania	Uganda
Sample size	1800	1800	1800	2000	900	1800	900
Geographic location (region, district [s])	Urban districts of Addis Ababa (Gulele, Yeka, Bole, Nefas Silk, Kirkos and Lideta)	Rural Zakiganj sub-district of Sylhet district, in northeast region	Peri-urban, coastal Karachi (Ibrahim Hyderi and Ali Akber Shah Goth) Sindh	Urban and peri-urban areas Sangam Vihar, Tigri, Madangir, Dakshinpuri and Khanpur in South Delhi	Ife community Ife Central, Ife Local Government Areas, Urban South west region, Osun state	All urban and rural districts in Pemba Island	Rural Iganga and Mayuge districts, Eastern region
Water, sanitation, socioeconomic status of study population	3% use unimproved water^a^ as main drinking water source, 46% use flush toilets. 93% have access to electricity. 9% women have no formal education (source Ethiopia DHS 2016)	21% use unimproved water as main drinking water source, 47% flush toilets. 87% have access to electricity. 15% women have had no formal education (source study area data)	14% use unimproved water as main drinking water source, 99% use flush toilets. 99% have access to electricity. 38% women have no formal education (source study area data)	No families use unimproved water as main drinking water source, 99% have access to a latrine; 95% are pit latrines and 5% have a flush tank. All have access to electricity. 15% women have no formal education (source study area data)	19% use unimproved water as main drinking water source (DHS 2018). 36% use flush toilets, 27% have access to electricity (MICS 2016). 6% women have no formal education (DHS 2018)	39% use unimproved water as main drinking water source, 10% use flush toilets. 22% have access to electricity 20% women have no formal education (DHS 2016 Tanzania [Pemba])	62% use unimproved water as main drinking water source, 1% use flush toilets. 17% have access to electricity (source study area data) 9% women have no formal education (DHS 2016 Uganda])
Wasting, underweight mortality 0–6 months	7.5% wasted, 14.3% underweight, Infant mortality 28 per 1000 live births	9.0% wasted, 22.6% underweight IMR 65 per 1000 live births	10.0% wasted, 23.5% underweight	9.3% wasted, 16.9% underweight Mortality 0–6 months 12 per 1000 live births	5.4% wasted, 16.2% underweight, Infant mortality rate 47 per 1000	5.3% wasted, 13.6% underweight Mortality 0–6 months 10 per 1000 live births	8% wasted, 16.2% underweight, infant mortality 53/1000 live births

^a^Unimproved water = unprotected dug well, spring, tanker truck, surface water

## Screening, eligibility criteria and enrolment

### Screening

Women are contacted between day 7 and day 14 post-partum and asked for informed written consent for their infants to be screened for eligibility for the trial. Screening takes place either at home or in health facilities after birth.

### Eligibility criteria

#### Inclusion criteria

- Ultrasound-based gestational age before 20 weeks of gestation is available
- Infant is born at 28 weeks gestation or more
- Birth weight is taken within 72 h after birth
- Infant has been fed breastmilk at any time since birth
- Infant age is between 7 days and less than 14 days
- Infant is a singleton or twin birth
- Informed consent has been obtained

#### Exclusion criteria

- Biological mother has died before eligibility screening
- Infant has a major abnormality or condition which is impairing feeding or nutrition (such as severe cleft palate, severe asphyxia, severe respiratory distress syndrome, need for mechanical ventilation, severe necrotising enterocolitis, major surgery)
- Family does not intend to live in the study area for 6 months
- Mother has given birth to three or more infants (that is, twins are included but triplets or quadruplets etc. are excluded)
- An infant has already been recruited and is under surveillance from the same household (that is, the infant is currently under active follow-up by the study team).

### Enrolment

Mothers of all eligible infants are asked for informed written consent for their infant to be included in the trial. The model informed consent form can be found in Appendix 2.

Strategies for maximising recruitment included community meetings and collaboration with local leaders and health providers to explain the study and answer any questions. Contingency plans to address low recruitment rates include extending the recruitment period and extending the geographic boundaries of the catchment areas. Mechanisms to maximise recruitment include explanation of the breastfeeding and clinical support the mother and infant is given and description of the incentives the mother is entitled to (soap and personal items for the baby).

## Randomisation

Eligible mother-infant dyads are randomised in a 1:1 ratio to the intervention (IBFCS plus NMS) or comparator (IBFCS alone) groups. The randomisation list has been prepared by an independent statistician using blocks of size 10.

Each site has a separate randomisation list, with additional stratification into eight groups according to the characteristics of the infant: singleton term appropriate for gestational age (AGA); singleton term SGA; singleton preterm AGA; singleton preterm SGA; multiple term AGA; multiple term SGA; multiple preterm AGA; and multiple preterm SGA. Both twins from a multiple pregnancy are allocated to the same trial group for ethical and logistical reasons. If there is discordance with regard to the AGA or SGA status of the twins, the SGA randomisation list is used.

Allocation to intervention and comparison groups is done through a server-based system, and the recruiters are not aware of the next allocation when recruiting infants. Allocation occurs when the mother-infant dyads are randomised (i.e. when the infant age is between 7 days to less than 14 days), and the allocation is stored within the infant’s database record. The allocation is only accessed if the infant is diagnosed with growth faltering. If an infant reaches study criteria for growth faltering the data management system triggers an alert to the study clinical team to assess the infant and to the intervention team to provide the intervention if the infant is in the intervention group (see Fig. 1). At this point, blinding of the supplementation team and the participants is not possible. However, all other members of the sponsor and the study teams including the IOA team will remain blinded as far as this is possible. The study statistics team will remain blinded throughout the trial and during the primary analysis of trial data.

## Baseline data collection

After randomisation, baseline anthropometric, health and sociodemographic data are collected from each mother-infant dyad when the infant is aged between 7 days and less than 14 days. The anthropometry (infant weight, length and head circumference) is done using standardised study equipment (weight using SECA 354 digital scales, length using SECA 417 infantometers and head circumference using SECA 212 head circumference measurement tapes) and procedures.

## Intensive breastfeeding counselling and support

Intensive breastfeeding counselling and support (IBFCS) is provided to all mother-infant dyads during the antenatal and postnatal periods by a dedicated team comprising a peer counsellor, breastfeeding counsellor and lactation consultant (see Table 3). The antenatal schedule includes three contacts at 28, 32 and 36 weeks gestation. The postnatal schedule includes two contacts in the first week after birth, then is weekly or two weekly until the infant reaches 6 months. The IBFCS visits use standard WHO guidance and tools [5, 13]. There are five components as shown in Table 4: antenatal and postnatal breastfeeding promotion, breastfeeding support, problem solving for breastfeeding difficulties and lactation management of more difficult problems. Additional support is provided if infant is premature, low birth weight or mother has breastfeeding problems. The visits occur at home or in a health facility wherever possible.

**Table 3:** Fieldwork teams

Peer counsellors Female, resident in the community for several years, with at least 10 years of schooling; preferably mothers with personal successful breastfeeding experience and motivated to help other mothers breastfeed. Peer counsellors will be trained using the adapted WHO Infant and Young Child Feeding Combined Course which includes provision of mental health support
Breastfeeding counsellors Female with formal training in health, at least 12 years of schooling and with prior training and experience in providing breastfeeding support including competency in supporting breastfeeding in preterm and small for gestational age (SGA) infants and with clinical/field experience in managing a small team, e.g. community health workers. Additional training on psychosocial issues is needed and training will be provided if the worker does not have these skills. Lactation counsellors will be trained in the WHO 40-h Breastfeeding Counselling Course
Lactation consultants Female senior health worker (may be a physician/senior nurse or midwife) who has received breastfeeding counselling training and has significant clinical/field experience including support of breastfeeding of preterm and SGA infants; also has proven experience in supervision of junior level staff. She will have had prior training in the WHO 40-h Breastfeeding Counselling Course and will receive additional Trainer of Trainer support. The term lactation consultant in the study is used to denote a higher level of skill and responsibilities. The training and position are not equivalent to that of the International Board of Lactation Consultant Examiners (IBLCE) certified lactation consultant
Clinical team Team of qualified nurses and doctors who have received training in IMCI and the WHO pocket book clinical management guidelines. At least one of the nurses or doctors is specialised in lactation management (called in this study a ‘lactation consultant’)
Supplementation team Team of qualified nurses and doctors who have received training in infant formula prescription, counselling and support

*WHO* World Health Organization, *IMCI* Integrated management of childhood illnesses, *SGA* Small for gestational age

**Table 4:** Activities in the intervention and comparator groups

Target population	Study group	Cadre of worker	Type of contact	Components
Whole population				
All pregnant study women during the antenatal period from 28 weeks gestation until birth	Intervention and comparator	Breastfeeding team	Breastfeeding promotion (antenatal)	WHO breastfeeding counselling guidelines on antenatal breastfeeding promotion: including promotion of early initiation of breastfeeding, prevention of separation of mother and infant, exclusive breastfeeding, attachment and responsive feeding, community support, anticipatory guidance [14, 15],
All mothers of study infants after birth	Intervention and comparator	Breastfeeding team	Breastfeeding promotion (postnatal)	WHO breastfeeding counselling guidelines on postnatal breastfeeding promotion: including early initiation of breastfeeding, prevention of separation of mother and infant, exclusive breastfeeding, attachment and responsive feeding, community support, anticipatory guidance, observing breastfeeding [15] WHO breastfeeding counselling guidelines on postnatal breastfeeding promotion for small babies for preterm and LBW babies including kangaroo mother care (KMC) [14]
Breastfeeding difficulties				
Mothers of study infants with breastfeeding difficulties	Intervention and comparator	Breastfeeding team	Breastfeeding support and problem solving for breastfeeding difficulties	WHO breastfeeding management guidelines including attachment, positioning, frequency of feeding, maternal health [15]
Growth problems				
Study infants with slow weight gain, growth concern or growth faltering	Intervention and comparator	Clinical team	Clinical assessment and management	Clinical assessment and management using WHO guidance for Integrated Management of Childhood Illnesses (IMCI) and WHO Pocket Book for Hospital Management of Children including history taking, physical examination and management of identified conditions [16, 17]
Study infants with slow weight gain	Intervention and comparator	Breastfeeding team	Additional breastfeeding support	WHO breastfeeding management guidelines including observing and assessing breastfeeding, problem solving including problems with a sleepy baby, attachment, positioning, frequency of feeding, maternal health [15]
Study infants with growth concern	Intervention and comparator	Breastfeeding team	Additional breastfeeding support	WHO breastfeeding management guidelines including observing and assessing breastfeeding, problem solving including problems with a sleepy baby, attachment, positioning, frequency of feeding, maternal health [15]
Study infants with growth faltering	Intervention and comparator	Breastfeeding team	Lactation management	Advanced WHO breastfeeding management guidelines including expression of breastmilk, cup or paladai feeding, scheduling of feeding, management of nipple problems, inverted nipples, mastitis, engorgement, maternal health and psycho social support and problem solving [15]
Study infants with growth faltering	Intervention and comparator	Clinical team	Regular clinical review	Regular clinical review by the clinical team using WHO guidance for Integrated Management of Childhood Illnesses (IMCI) and WHO Pocket Book for Hospital Management of Children including history taking, physical examination and management of identified conditions [16, 17]. The review occurs on day 1, 3, 7 and weekly until the recovery point. If there is deterioration or concerns the infant is discussed with each site’s senior clinical team and referred to hospital where needed
Study infants with growth faltering	Intervention and comparator	Clinical team	In depth clinical case review	In depth case review if growth faltering every 4 weeks until the recovery point. The clinical team assesses if the infant has reached the target weight and if the infant has not reached the target weight then the possible reasons for this and counsels the mother accordingly. The clinical team uses WHO guidance for Integrated Management of Childhood Illnesses (IMCI) and WHO Pocket Book for Hospital Management of Children including history taking, physical examination and management of identified conditions. If there is deterioration or concerns, the infant is discussed with each site’s senior clinical team and referred to hospital where needed
Study infants at recovery point	Intervention and comparator	Clinical team	In depth clinical case review	When the infant reaches the recovery point, a final in depth case review is performed and then the infant resumes normal growth monitoring and surveillance
Supplementation (intervention delivery)				
Study infants with growth faltering	Intervention group only	Supplementation team	Supplementation	Provision of the nutritional milk supplement (NMS) intervention (prescribed quantities of term infant formula that meets Codex Alimentarius standards and calculated to meet the needs for catch up growth) by the mother to the infant after counselling on responsive feeding and hygiene standards Standardised study SOPs are used to counsel the mother about giving the NMS hygienically to the infant using the responsive feeding method. The infant’s cues are observed. The infant is first put to the breast and supported to breastfeed. The NMS is then offered after breastfeeding [14]. The infant is offered NMS at least 6 times per day The infant receives regular clinical review and 4 weekly in depth clinical case review as described above. The NMS review visits occur immediately after the clinical reviews The data management system calculates the amount of NMS to be offered to the infant to meet the infant’s caloric needs for catch up growth. Every 4 weeks the data management system recalculates the amount of NMS to be given to the infant and the mother is counselled about the new management plan
Study infants at recovery point	Intervention group	Supplementation team	In depth clinical case review	When the infant reaches the recovery point, a final in depth case review is performed as described above. In the intervention arm, it is also explained to the mother that the infant can cease the NMS. If there are any concerns about stopping the NMS, a case review meeting is held with the senior site clinicians and senior clinicians in the sponsor team. This senior team decides the NMS plan based on the best interests of the infant. If the NMS is continued, a protocol deviation form is completed and the infant continues the regular clinical reviews and in depth reviews as described above. The future management plan is adjusted based on these reviews

## Infant surveillance and growth monitoring

*Infant surveillance* is conducted by a designated IOA team. The visits are conducted weekly until the infant reaches 4 weeks of age and then fortnightly until the infant reaches 6 months of age. The schedule is listed in the activity matrix in Fig. 2.

At each surveillance visit, data are collected on infant health status including vital status (alive or dead), episodes of diarrhoea, respiratory infection, other infections, other illnesses and hospitalisations. Data are also collected on infant feeding practices including breastfeeding, exclusive breastfeeding and other intake of fluids or foods. Mothers are asked how they have fed their baby since the last visit and in the last 24 h.

**Fig. 3:**
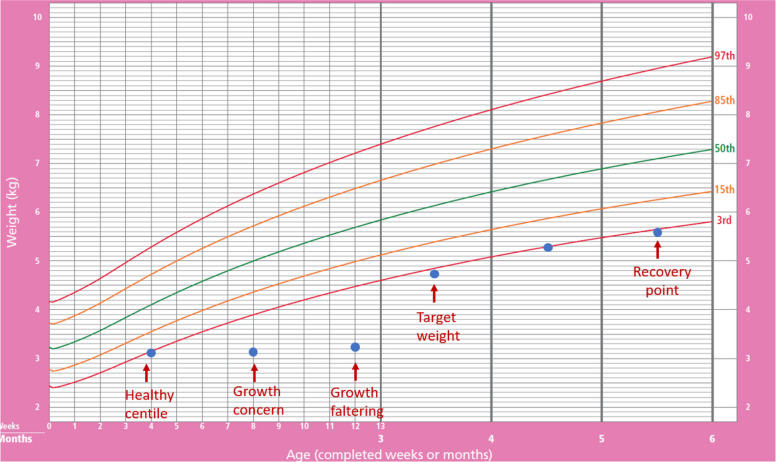
Growth monitoring schema

Growth monitoring. At each visit, the infants are also weighed by the IOA team using standardised weight scales (SECA 354 digital scales). The weight measurements are then inputted into the data management system and compared to growth reference standards (see below), and infants are categorised as follows: healthy centile, slow weight gain, growth concern, growth faltering, reached target weight or reached recovery point (see Table 5 and Fig. 3).

**Table 5:** Growth phases and definitions in the trial

Phase	Measurement method	Definition
Healthy phase		
Healthy centile	Change in weight in grams over 2 weeks	Centile before slow weight gain was identified
Precursor phase		
Slow weight gain*	Change in weight in grams over 2 weeks	If WFA < 3rd centile: fall in weight velocity > 15th centile
		If WFA > = 3rd centile: fall in weight velocity > 25th centile
Growth problems		
Growth concern*	Change in WFA over 4 weeks	If WFA < 3rd centile: any fall in weight for age centile
		If WFA > = 3rd centile to < 15th centile: fall of 1 or more centile spaces
		If WFA > = 15th centile to < 85th centile: fall of 2 or more centile spaces
		If WFA > = 85th centile: fall of 3 or more centile spaces
Growth faltering*	Change in WFA over 4 weeks	If WFA < 3rd centile: any fall in weight for age centile
		If WFA > = 3rd centile: fall of one or more centile spaces
Recovery phase		
Target weight	Weight in grams	Weight in grams the infant will reach in 28 days (4 weeks) if the infant grows along the healthy centile
Recovery point	Weight in grams	Weight in grams the infant will reach if the infant grows along the healthy centile for 56 days (8 weeks) after the target weight is reached

Weight velocity = weight gain in grams per day

*WFA* = weight for age

Calculations assume one centile space = 0.33 z scores and that a centile space equals the space between adjacent centile lines, 1–3, 3–5, 5–15, 15–25, 25–50, 50–75, 75–85, 85–95, 95–97, 97–99

Calculations use WHO reference standards for term infants or Intergrowth reference standards for preterm infants

^*^Measured over 28 days for growth concern and growth faltering; and over 14 days (7 days if < 4 weeks of age) for slow weight gain

Data are collected during home visits. If the infant cannot be located at home, surveillance data are collected by phone if needed.

## Definition and management of growth problems

Tables 4 and 5 provide details of the definitions and management of growth problems in infants in the trial.

### Healthy centile

The ‘healthy centile’ is defined in this trial as the centile the infant’s weight has been following since birth before any growth problems are identified.

### Slow weight gain

‘Slow weight gain’ is defined by comparing the difference in weight over a 2 week period to the WHO growth velocity reference standards for term infants [18]. If an infant is < 3rd centile and has a fall in growth velocity of > 15th centile or if the infant is > 3rd centile and has a of > 25th centile, the infant is classified as having slow weight gain.

All mothers of infants with slow weight gain receive IBFCS. All infants with slow weight gain are reviewed by study clinicians and their medical problems are treated. All infants with slow weight gain are reviewed at least every 2 weeks. Details can be found in Tables 4 and 5.

### Growth concern

‘Growth concern’ is defined by comparing the difference in weight for age (WFA) z score/centile over a 4 week period to WHO growth reference standards for attained weight (weight for age, WFA). WFA is compared to the WHO growth reference standards for term infants [18] or intergrowth growth reference standards for preterm infants [19]. Infants are defined as having growth concern if they fulfill any of the following conditions: if an infant has WFA < 3rd centile at the beginning of the month and then has any fall in weight for age centile, if WFA > = 3rd centile to < 15th centile and then has a fall of one or more centile spaces, if WFA > = 15th centile to < 85th centile and then has a fall of 2 or more centile spaces, if WFA > = 85th centile and then has a fall of 3 or more centile spaces. In the data management system, a one centile space fall is defined as greater or equal to a 0.33 z scores [2, 3]. These cut points were derived from WHO [2] and the United Kingdom National Institute Centre of Excellence (UK NICE) growth faltering guidelines [14].

All mothers of infants with growth concern receive additional IBFCS from a breastfeeding counsellor. All infants with growth concern are reviewed again by study clinicians and any medical problems are treated. All infants with growth concern are reviewed at least every 2 weeks. Details can be found in Tables 4 and 5.

### Growth faltering

Growth faltering is defined as a further drop in z score/centile in the next month after growth concern is identified. If WFA < 3rd centile: any fall in centile; if WFA > = 3rd centile: fall of one or more centile spaces. These cut points were also derived from WHO [2] and the United Kingdom National Institute Centre of Excellence (UK NICE) growth faltering guidelines [14].

At this point, all mothers of infants with growth faltering receive additional IBFCS from a lactation consultant. All infants with growth faltering are reviewed again by study clinicians and any medical problems are treated. All infants with growth faltering are reviewed on day 1, day 3 and day 7 and then weekly until they improve. Every 4 weeks, there is an in depth clinical case review of each infant by the senior clinical team in each site. Details can be found in Tables 4 and 5.

If the infant is in the intervention arm, they also receive nutritional milk supplementation (NMS) as described below.

### Target weight

The infant’s target weight is defined as the weight in grams the infant will reach in 28 days (4 weeks) if the infant grows along the healthy centile.

### Recovery point

The infant’s recovery point is defined as the weight in grams the infant will reach if the infant grows along their healthy centile for 56 days (8 weeks) after the target weight is reached. At the recovery point, the infant has an in depth case review, and the mother is advised that the infant can resume normal growth surveillance, normal breastfeeding support schedules and cease all other support (e.g. the clinical reviews and NMS are stopped at this time).

### Red flags

Red flags are defined as follows: (i) an infant with growth faltering who falls additional centiles after 4 weeks of treatment, (ii) an infant with growth faltering who has no improvement in growth after 4 weeks of treatment, (iii) an infant with a weight for length z score (WLZ) < −3 standard deviations (SD) (severe wasting), (iv) an infant who the study staff or the mother have concerns about.

Study staff discuss all infants with red flags with senior clinical staff and assist the infant to receive hospital care as needed. Referral and transport to health facilities is facilitated for all infants who require assistance.

## Intervention and comparison groups

### Comparison group

All infants in the comparison group who meet the criteria for growth faltering receive the schedule of IBFCS, clinical care, treatment of medical problems, infant surveillance and growth monitoring described above but do not receive the study NMS.

### Intervention group

All infants in the intervention group who meet the criteria for growth faltering receive the schedule of IBFCS, clinical care, treatment of medical problems, infant surveillance and growth monitoring described above plus the intervention: nutritional milk supplementation (NMS). The NMS is delivered by the intervention delivery team (called in this study the supplementation team).

#### Intervention details

The intervention (NMS) is Codex Alimentarius standard term infant formula [20, 21]. It is equivalent in nutritional composition to term infant formula and has been manufactured according to Codex Alimentarius and Good Manufacturing Practices (GMP), and all nutritional content is compliant with the specifications [20, 21]. The NMS in six sites was procured by UNICEF until December 2025, and manufactured by GeoPoland, Warsaw, Poland [4]. In one site, prior to December 2025, and in six sites, from January 2026, the NMS is Codex compliant standard term infant formula procured incountry, In accordance with the manufacturer’s instructions, the NMS is stored in all site offices at less than 30 °C.

The amount of NMS offered to the infant is calculated to meet the United Nations Food and Agricultural Organization (FAO) nutritional (energy, protein, macronutrient, micronutrient) requirements for catch up growth of the infant [22]. This calculation divides the infant’s target weight by the infant’s current weight and multiplies by 100 kcal/kg/day to give a total volume in mls per kg per day. If the infant is considered to be fully breastfed, the calculation will subtract 100 kcal/kg/day. The data management system then calculates the amount in mls to give to the baby per day. The baby is offered the NMS feeds at least six times a day so the data management system divides the daily volume by six to provide the amount in mls to offer the baby at each feed.

Each mother is given canisters of NMS by the field workers at home. At the first visit, mothers are counselled by the research staff in the correct use of NMS including using WHO standard instructions on the correct amount of NMS and water, hygienic preparation and the methods of feeding [15]. Mothers also receive counselling about the importance of hand and equipment washing, boiling water and using towels for mopping up spillage on the infant. Mothers are given feeding demonstrations and asked to prepare and administer feeds directly observed by research staff. Mothers are advised not to use bottles or teats. Mothers are counselled to use locally available items for preparation. If the mother does not have a feeding cup, spoon or container to mix up the NMS, these are offered to the mother by trial staff. No bottles and teats are procured by the sites or offered to the mother.

The NMS is provided to the infant using the responsive feeding method [16]. Responsive feeding is defined according to WHO and UNICEF as recognising the infants’ cues for feeding, closeness and comfort, and responding accordingly to these cues [16]. The baby is offered the NMS feeds at least six times and whenever the baby is cueing or appears hungry after a breastfeed. Mothers are trained and counselled in the responsive feeding method by the supplementation team using WHO procedures [16]. The baby’s cues are followed at all times. The baby is not force-fed. At each feed, the baby is encouraged to breastfeed first before any attempt at feeding NMS is made, i.e. the baby is put to the breast first and breastfed before offering the calculated amount of NMS. If the baby is cueing and still appears hungry after the NMS is finished then the baby is offered additional NMS.

Any NMS not consumed at the time of the feed is discarded after 2 h in line with published WHO guidance and a fresh batch made up for each feed [15]. After each use, the mother is advised to store the NMS canister in the family’s home in the coolest driest environment available.

Every 4 weeks, the data management system recalculates the amount of NMS to be given to the infant, and the mother is counselled about the new management plan. Further details can be found in Table 4.

#### Monitoring adherence

At each follow up visit, the mother is asked to show the NMS canister and the supplementation team records the amount remaining in the canister, if there is any contamination or damage, and the number of canisters used. The mother is also asked how many times she has fed the NMS in the last 24 h and if she fed the prescribed amount of NMS.

#### Recovery point activities

At the recovery point, an in depth case review about the NMS use is done. The mother is informed that the NMS can be stopped. If there are any concerns at this point, an additional case review meeting is held with the senior site clinicians and senior clinicians in the sponsor team. After the NMS is stopped, the supplementation team collects all NMS canisters from the family regardless of the amount of NMS powder in the canisters and returns and logs them back into the office.

### Participant retention and adherance to the interventions

Strategies for promoting participant retention include the regular 1–2 weekly in person follow up visits and relationship building. Strategies for ensuring adherence to the interventions include the counselling from study staff at each visit (1–2 weekly) about the importance of the interventions such as IBFCS and the use of the NMS (in the intervention arm). Adherance to the study interventions is monitored by the study staff recording: the number of visits conducted and any difficulties conducting the visits; the mother’s current breastfeeding practices; the number of NMS canisters used and the amount of milk powder remaining in the canisters (if in the intervention arm). If participants discontinue any part of the study interventions, they are encouraged to remain in the other parts of the study and to be followed up for complete outcome data collection by the IOA team. As discussed in the data analysis section, participant data will be analysed by intention to treat in the primary analysis.

### Co-interventions and concomitant care

At all times, the families of all infants in both the intervention and the comparator groups may seek care for their infant from any health care provider. All careseeking, medical care or additional nutritional supplements given are documented on the study CRFs at the IOA team visits. These data will be compared between the intervention and comparator groups in the final data analysis.

## Safety monitoring

In this trial, a serious adverse event (SAE) is defined according to International Council For Harmonisation Of Technical Requirements For Pharmaceuticals For Human Use-Harmonised Guideline For Good Clinical Practice (ICH-GCP) [17] as any unfavourable medical occurrence that is considered serious at any dose if it results in (i) death, (ii) requires inpatient hospitalisation, (iii) is life-threatening, (iv) results in persistent or significant disability/incapacity, (v) requires prolongation of existing hospitalisation or (vi) is a congenital anomaly/birth defect.

At every visit from the IOA team and clinical team, the infant’s mother is asked about any infant illnesses using a standardised CRF. All SAEs are notified to the WHO TCU within 24 h of identification. All SAEs are graded by the trial sponsor according to ICH-GCP including their relatedness to the NMS provision. If the study staff have any concerns about an illness that they think may be related to the NMS at any time, the NMS will be stopped immediately. The principal investigators, WHO and the DSMB will be informed and a full investigation will be conducted. All infants with SAEs are assisted to reach the necessary medical care and all SAEs are followed up by the study principal investigators until resolution. SAEs are also reported according to site and WHO requirements.

## Protocol deviations

A protocol deviation (PD) is defined as any part of the trial that is in non-compliance with the trial protocol, SOPs or ICH GCP R2. Protocol deviations may be identified in the field, by the principal investigators, TCU data management team, the external monitor or another team member. PDs are defined according to ICH-GCP [17] as critical, major or minor. All critical or major PDs are notified to the sponsor within 24 h; all minor PDs are reviewed by site principal investigators and the sponsor each month. PDs are reported to regulators and ethics committees as per the site and sponsor requirements.

## Data management and analysis

### Data management

WHO/NBC is the data controller and custodian for this trial. Data are managed centrally by a clinical trials data management team, supervised directly by the sponsor. A web-based, GCP-compliant data management system is used. Data are collected electronically using electronic tablet interfaces. Range and logic checks are built in to ensure data quality. Real time data are transferred to local and web-based servers. The site principal investigators monitor their data in real time and weekly and send monthly data to the TCU. The TCU monitors data monthly including logic errors and checks across different forms. Queries generated are given to study teams for resolution and corrections incorporated.

This trial is generating an anonymised research dataset, with de-identified information of characteristics and outcomes from all women and infants participating in the trial. Each site has access to its own site data during the trial to facilitate self-monitoring by the site team. To ensure confidentiality, data are anonymised of any identifying participant information. Identifiable data are retained until the files are clean, analysis completed and results published. De-identified data will be stored permanently. If a mother decides to withdraw from the study, the researchers ask for permission for the mother’s data and her baby’s data to be retained in the databases. If the mother refuses then the researchers delete it from the study databases. After completion of the trial, the trial’s documents will be archived in accordance with institutional and national rules for clinical research archiving.

### Sample size calculations

The full details of the sample size calculations can be found in the statistical analysis plan (SAP) in Appendix 4. The following assumptions have been used for sample size calculations:

- Mortality between enrolment and 6 months of age will be 1–2% and wasting at 6 months among survivors will be 6–8% in the comparison group. The primary outcome will therefore be 9% (range 7 to 10%) in the comparison group
- The hypothesized primary outcome in the intervention group will be 20% lower compared to the comparison group, i.e. 7.2% infants (range 5.6 to 8.0%)
- 95% confidence level, 90% power
- 10% loss to follow up during the study period

With the above assumptions, the total sample is 11,000. A total of 11,000 infants will therefore be enrolled across seven study sites as shown in Table 2.

### Data analysis

The full details of the data analysis can be found in the statistical analysis plan (SAP) in Appendix 4. The primary analyses will be pooled across all sites. Analyses will be conducted on an ‘intention-to-treat’ basis, with infants analysed in the group into which the mother-infant dyad was randomised, irrespective of whether participants received NMS (if randomised to this group) or the ‘dose’ received.

#### Descriptive statistics

To understand the characteristics of site level populations, data on mortality, morbidity, growth, infant feeding patterns and careseeking (including treatments received) in the first 6 months of life will be summarised for the whole population and among infants who reach growth faltering. The flow and number of infants through assessment of eligibility, randomisation, completeness of follow-up and analysis will be presented, along with reasons for exclusions and withdrawals. Baseline characteristics of infants and their mothers and families will be compared in the intervention and comparator groups to assess whether randomisation has achieved adequate balance in these characteristics between the groups. Summary values (means, proportions) for infant, maternal and household characteristics in the intervention and comparison groups will be presented. Significance tests will not be done.

#### Comparative analyses

The primary analysis will assess the effect of the intervention compared with the comparison group on the binary outcome, wasting-free survival at 6 completed months of age. The intervention group will be compared against the comparison group for the primary outcome using unadjusted and adjusted risk ratios with 95% confidence intervals (CI). To account for the potential clustering from twin births and repeated pregnancies in the same woman, generalized estimating equations with a logit link will be used for the adjusted analyses, with the mother included as a cluster and all of the other factors included in the stratified randomisation (site, AGA or SGA, term or preterm, singleton or multiple birth) included as covariates. The QIC (quasi-likelihood under the independence model criterion) will be used to choose between an independence, exchangeable and unstructured correlation structure [23]. The resulting estimated coefficients (on the log odds scale) will be converted to risk ratios via the predicted risks and the uncertainty about the resulting risk ratios estimated using the delta method. The primary analysis will be a complete case analysis (including only infants with complete outcome data) if there is less than 5% missing data on the primary outcome, or if we are unable to identify predictors for the missingness. If a complete case analysis is not appropriate, we will account for the missing data using multiple imputation, with the missing data assumed to be missing at random.

Analysis of the secondary outcomes will assess the effect of intervention on mortality, wasting, severe wasting, underweight, concurrent wasting and stunting, and morbidity, measured at 6 completed months of age, and breastfeeding practices will be measured at 5 completed months. In addition, we will assess and report the proportion of infants in the intervention group who receive the trial NMS. The effect of interventions on binary secondary outcomes will be assessed using the same models as for primary outcomes. For continuous outcomes, means and standard deviations will be presented for all z scores and morbidities. Generalized linear models of the Gaussian family with an identity-link function will be used to estimate the effect sizes (difference in means and 95% CIs).

#### Sub-group analyses

We will conduct subgroup analyses for the effects of the intervention:

- By gestational age and birth weight in four groups: term AGA, preterm AGA, term SGA, preterm SGA;
- By infant sex;
- By study site (individually and whether the site is in Africa or Asia); and
- In growth faltered and non-growth faltered infants separately.

The relative measures of effect within each of these subgroups will be estimated. We will conduct a test of homogeneity of effects across the subgroups and report a *p* value. Unless there is strong evidence against the null hypothesis of homogeneity of effects (i.e. *p* < 0.001), the overall risk ratio will be considered the most useful guide to the approximate relative risks in all subgroups.

#### Sensitivity analyses

The following sensitivity analyses have been pre-specified:

-Including only infants who have remained in the trial for at least 7 days after growth faltering has been identified;
-Including only infants who have remained in the trial for at least 14 days after growth faltering has been identified;
-Excluding critical protocol deviations (as defined above);
-Using different definitions of infant mortality (infant mortality defined as mortality to 182 days only; and defined as mortality to the end of the follow up period of 209 days);
-Using different definitions of careseeking (allowing for a wider definition of healthcare providers in accordance with the different systems of care that exist in the sites);
-Additional adjustment (that is, in addition to the adjustment for stratification variables already specified above) if there are imbalances in other variables that may influence the primary outcome. Whether the variables are likely to influence the primary outcome and the level of imbalance which would trigger adjustment will be discussed with domain experts and the principal investigators at the time of the analyses, with all decisions reported clearly in any publications of the results.

## Ethical approval and consent to participate

Ethical approval has been obtained from the ethical review committees of all sites and WHO (see Appendix 7). Written individual informed consent in the local languages is obtained from all mothers prior to their inclusion into the trial (i.e. prior to reproductive surveillance, pregnancy surveillance, and infant enrolment). A research team member approaches each mother to ask for consent. Each mother is given a copy of the information sheet and is allowed time to read the sheet. The researcher reads out the form if the mother is unable to read or would like assistance. The researcher also asks the mother if she understands the information in the form. If the mother appears not to understand any parts of the form, the researcher explains each sentence until it is clear the mother understands. If mothers are unable to sign, a thumb imprint is taken and witnessed by an impartial literate witness.

The study pays for any test that is performed by the research team and any adverse events due to the NMS. Compensation for time spent during study procedures is provided according to site specific protocols (non-monetary compensation, e.g. soap, phone credit, baby gift, utility items) in all sites.

This study involves participants and their families as partners in research. The site teams have long standing links in the study areas and with the local leaders, villages, towns and cities. As per usual procedures, the local principal investigators have held meetings with local leaders to discuss the best methods of community engagement, information sharing and dissemination. Local meetings are held to explain the study and to ask for input and also for question and answer sessions. The site teams will also conduct end of study dissemination activities in each site.

Study procedures are complementary to existing health services. Mothers and families are encouraged to attend their own health facilities for health services. The study teams explain to mothers that they must seek care when they are concerned about health problems with themselves and their baby. The study teams also educate mothers about the important danger signs that require careseeking. The study teams ask families to inform them when they are seeking care from health facilities so they can facilitate referral.

All protocol amendments are notified to all trial ethics committees and trial registries and journals. All SAEs and protocol deviations are notified to all WHO and site ethics committees and regulators according to sponsor and site level procedures.

## Training and standardisation

Prior to study initiation, all staff were trained in the study objectives, study strategy and in good clinical practice (GCP). Additionally, each team has undergone intensive training in their area of work (surveillance, consenting, anthropometry measurements, assessment of morbidity, IBFCS, clinical care, provision of supplements). Inter- and intra-observer standardization exercises for anthropometry are conducted and repeated every 6 months during the study implementation period. Weighing scales and length measurement instruments are routinely calibrated at least weekly using standard weights and length rods. Eligibility criteria for selection of staff can be found in Appendix 6.

## Monitoring and quality assurance

Each of the study teams has their own supervisor who train and monitor their teams and support adherence to the study SOPs. This includes scheduled and unscheduled visits, review of skills and quality of data collected, and feedback and supportive supervision.

The sponsor also monitors weekly status reports meet with each site on a monthly basis and conducts regular site visits. Additional periodic review meetings are also organized between the study teams, coordinators and the investigators. The sponsor also conducted site preparation reviews before the initiation of the trial. This included standardization of practices and measurements.

There is an external trial monitor who performed all site activation procedures and monitors the trial. The TCU will organise audits as and when required. These will be independent of the sites and the sponsor.

## Discussion

This large randomised trial will provide evidence about the role of NMS, if any, in infants with growth faltering who do not respond to IBFCS and treatment of medical problems in low resource settings.

There are no current WHO guidelines on managing growth problems in the first 6 months of life, and to our knowledge, no previous trials have addressed this issue. The results of this trial will help close this evidence gap. Importantly, the findings from this trial will be used to develop WHO recommendations to improve management of infants under 6 months of age with growth problems. The findings will also be used to develop national clinical guidelines and community level health care protocols for LMICs. It is also anticipated that secondary analyses of trial data will also be conducted and published.

The investigators are committed to the widespread dissemination of the findings of this study. This will follow a process of sharing results with the participants and local health care providers, Ministry of Health staff and the international community. Firstly the study teams will share the findings with the study participants through meetings with their local health care providers and community meetings.

The study teams will discuss and disseminate results in each site as soon as analyses are finished and the first reports are completed. Derivative products (including evidence summaries, policy briefs and other tools to facilitate implementation) will be developed and widely disseminated. The primary trial findings will be prepared as a peer-reviewed manuscript, and published in an open-access, international journal and widely disseminated.

A breastfeeding-led nutritional strategy for managing growth faltering and learning how to implement it will have important benefits in reducing vulnerability and increasing the resilience of infants. This study will provide important information on how to effectively deliver quality breastfeeding and support for the general population and especially for high- risk young infants such as those with low birth weight or preterm which is essential for scale up into existing health systems.

## Trial status

Recruitment for the BRANCH trial started in September 2024 and is currently ongoing (expected to be completed by June 2027). This manuscript is based on the current version of the protocol, version 1.4, 8 July 2024 which was approved by the WHO Ethics Review Committee in July 2024.

## Supplementary Information

Additional file 1: Appendix 1. Trial field work schema

Additional file 2: Appendix 2. Model informed consent form

Additional file 3: Appendix 3. SPIRIT check list

Additional file 4: Appendix 4. Statistical analysis plan

Additional file 5: Appendix 5. Criteria for selecting sites

Additional file 6: Appendix 6. Criteria for selecting personnel

Additional file 7: Appendix 7. WHO ethical approval

Additional file 8: Appendix 8. Funding information

## Data Availability

WHO (the sponsor) has access to the final trial dataset; data will be available at the end of the trial on application to the trial sponsor. All relevant materials are available on reasonable request from the corresponding authors.

## Abbreviations

AE: Adverse event
BRANCH: Acronym for the trial, BRANCH, *B*re*a*stfeedi*n*g *c*ounselling and ma*n*agement of growt*h*
CRF: Case record form
DSMB: Data safety and monitoring board
EAG: External advisory group
ERC: Ethics review committee
GMP: Good manufacturing practice
IBFCS: Intensive breastfeeding counselling and support
ICF: Informed consent form
IOA: Independent outcome assessment
ICH-GCP: International council for harmonisation of technical requirements for pharmaceuticals for human use harmonised guideline. Good clinical practice (ICHGCP)
LBW: Low birth weight
MCA: WHO Department of Maternal, Newborn, Child, and Adolescent Health & Ageing
MUAC: Mid upper arm circumference
NBC: Newborn and Child Health and Development Unit
SAE: Serious adverse event
SOP: Standardised operating procedure
SD: Standard deviation
TCU: Trial coordinating unit
TOR: Terms of reference
WAZ: Weight for age z score
WLZ: Weight for length z score
WHO: World Health Organization
