# Correction: Nutritional management of growth faltering in infants aged under six months in Asia and Africa: study protocol for a multicentre randomised trial (BRANCH, *BR*e*A*stfeedi*N*g *C*ounselling and management of growt*H*)

**DOI:** 10.1186/s13063-026-09667-7

**Published:** 2026-03-27

**Authors:** WHO BRANCH study group

**Affiliations:** Organisation mondiale de la Sante, Geneva, Switzerland

**Correction: Trials 26, 492 (2025)**

**https://doi.org/10.1186/s13063-025-09034-y**

Following the publication of the original article [1], we were notified of one change needed in the following paragraph as UNICEF is not able to provide a second batch of milk supplement.

Originally published paragraph:

“The NMS in six sites has been procured by UNICEF and manufactured by GeoPoland, Warsaw, Poland [4]. Due to country regulations, the NMS in one site is Codex compliant standard term infant formula procured incountry. In accordance with the manufacturer’s instructions, the NMS is stored in all site offices at less than 30 ℃.”

Corrected paragraph:

“The NMS in six sites was procured by UNICEF **until December 2025**, and manufactured by GeoPoland, Warsaw, Poland [4]. **In one site, prior to December 2025, and in six sites, from January 2026**, the NMS is Codex compliant standard term infant formula procured incountry, In accordance with the manufacturer’s instructions, the NMS is stored in all site offices at less than 30 ℃.”

The original article has been corrected.
