# Supplementary material for: Nutritional management of growth faltering in infants aged under six months in Asia and Africa: study protocol for a multicentre randomised trial (BRANCH, BReAstfeediNg Counselling and management of growtH)
Source: Trials. 2025 Nov 6;26:474. doi: 10.1186/s13063-025-09034-y (PMC12590774; doi:10.1186/s13063-025-09034-y)

**Title - Nutritional management of growth faltering in infants aged under six months in Asia and Africa. Study protocol for an individually randomised trial (BRANCH, BReAstfeediNg Counselling and management of growtH)**

**Authors – WHO BRANCH study group**

**Version date – 1Aug2025**

**Appendix 1. Trial field work schema**


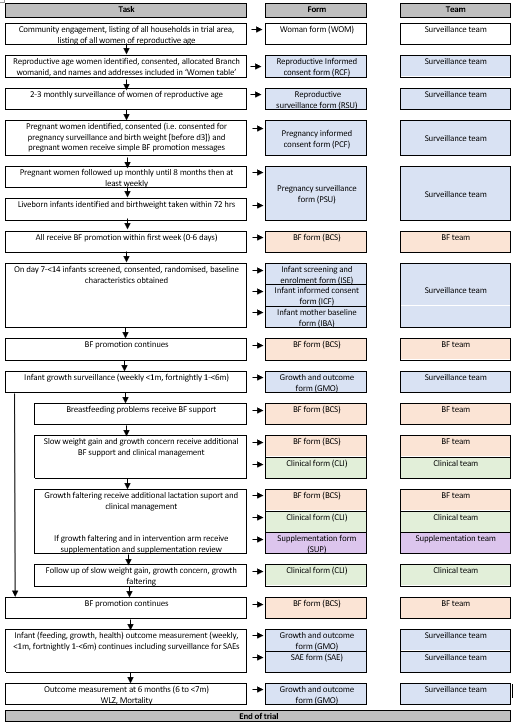

Supplement: Supplementary file 1 — Additional file 1: Appendix 1. Trial field work schema [file 13063_2025_9034_MOESM1_ESM.docx]
