# Supplementary material for: Nutritional management of growth faltering in infants aged under six months in Asia and Africa: study protocol for a multicentre randomised trial (BRANCH, BReAstfeediNg Counselling and management of growtH)
Source: Trials. 2025 Nov 6;26:474. doi: 10.1186/s13063-025-09034-y (PMC12590774; doi:10.1186/s13063-025-09034-y)
Supplement: Supplementary file 2 — Additional file 2: Appendix 2. Model informed consent form [file 13063_2025_9034_MOESM2_ESM.docx]

**Title - Nutritional management of growth faltering in infants aged under six months in Asia and Africa. Study protocol for an individually randomised trial (BRANCH, BReAstfeediNg Counselling and management of growtH)**

**Authors – WHO BRANCH study group**

**Version date – 1Aug2025**

**Appendix 2. Model informed consent form**

**Nutritional management of growth faltering in infants aged under six months in Asia and Africa. An individually randomised trial. Informed consent form. Version 1.4. 8July2024**

**INFORMATION SHEET FOR WOMEN AND INFANTS IN MAIN STUDY**

This information sheet is for women who have just given birth who we are inviting to participate in research on growth in infants aged under 6 months.

The title of our research project is “Nutritional management of growth faltering in infants aged under six months in Asia and Africa. An individually randomised trial*”*

**[Name of Principal Investigator]** *[site specific details]*

**[Name of Organization]** *[site specific details]*

**[Name of Sponsor]** World Health Organization

**[Name of Proposal and version]**  Nutritional management of growth faltering in infants aged under six months in Asia and Africa. An individually randomised trial. Version 1.4. 8July2024

This Informed Consent Form has two parts:

- Information sheet (to share information about the research with you)
- Certificate of consent (for signatures if you agree to take part)

**PART I: Information Sheet**

**Introduction**

I am [*research staff name*], working for *[research organization, site specific details]*. We are doing research to find out the best ways to feed babies who have problems with growth in the first six months after birth. Problems with growth are very common in this country. I am going to give you information and invite you to be part of this research. You do not have to decide today whether or not you will participate in the research. Before you decide, you can talk to anyone you feel comfortable with about the research.

There may be some words that you do not understand. Please ask me to stop as we go through the information, and I will take time to explain. If you have questions later, you can ask them of me or our other research team members.

**Purpose of the research**

We are doing research to find out the best ways to feed babies who have problems with growth in the first six months after birth, which is very common in this country.

The reason we are doing this research is to find out, for babies with growth problems, if nutritional supplementation plus intensive breastfeeding support is better than intensive breastfeeding support alone.

**Participant selection**

We are inviting all new mothers, whose babies are between seven and fourteen days old, to be part of this study.

**Voluntary participation**

Your and your baby's participation in this research is entirely voluntary. It is your choice whether to participate or not. Whether you choose to participate or not, you and your baby will still receive the same care and attention by *[research organization, site specific details]* and the health facilities in the study area. You may change your mind later and stop participating even if you had agreed to do so earlier.

**Study procedures**

If you agree for you and your baby to participate in this research study, then:

- Your baby will be placed in one of the two groups as if by lottery which is similar to tossing a coin. If your baby falters in growth, depending on which group your baby is in, the baby will either receive intensive breastfeeding support plus nutritional supplementation or intensive breastfeeding support alone without nutritional supplementation.
- We will ask you some questions about your family and living conditions
- Between day seven and fourteen after birth we will measure your baby’s weight, length, and head circumference.
- We will visit your home weekly until your baby reaches 4 weeks of age and then 2 weekly. This will be to find out how your baby is progressing. At these home visits, we will measure your baby’s weight and will ask you about how you are feeding your baby and any illnesses and visits to health services that your baby has had. We will also ask if your baby has had runny stools (diarrhoea) and fast breathing (pneumonia).
- We will contact you and arrange convenient times to visit and offer breastfeeding support until your baby reaches 6 months
- If you are having problems feeding your baby or if your baby has problems with growth and weight gain we will offer you additional support from breastfeeding experts, nurses and doctors. If you give permission, you will be visited at home or invited to come to the health facility. The breastfeeding experts, nurses and doctors will examine you and your baby and offer you advice. If you or the baby need it and if you give permission, the doctors and nurses may do tests on the baby or yourself. We will continue to review you and your baby regularly until you are both recovered.
- If your baby has continued problems with growth and weight gain and is in the nutritional supplementation group, we may offer you a nutritional milk supplement along with continued breastfeeding support. We will explain to you why your baby needs the supplement and we will show you how to prepare it, how much to give your baby, and how to keep it clean. We will continue to give you this nutritional milk supplement and to review your baby regularly until your baby has started to gain weight again.
- If your baby is in the control arm, and shows signs of growth faltering, we will continue to provide intensive breastfeeding support, but no nutritional milk supplement will be provided by the study team.
- When the baby reaches 6 months of age, we will measure your baby’s weight, length, head circumference, and upper arm circumference.

**Duration**

Your baby will be in the study for 6 months. During that time, you will be visited at home. There will be no need for you to come to the health facility unless the baby is unwell. Each visit should not be more than 30 minutes.

**Risks and discomforts**

Use of the nutritional milk supplement may carry risks. You have to be careful in preparing the nutritional milk supplement so you give the baby the correct amounts and so it doesn’t get contaminated. Giving the wrong amounts e.g. dilution or preparing the milk in an unhygienic way can result in illness in the baby, so it is important to follow the instructions our team will provide to you to prepare the nutritional milk supplement. We will show you how to prepare the nutritional milk supplement and how to give it to your baby so your baby doesn’t get sick and will answer all your questions.

You may become tired of us visiting you at home. If at any time during the study, you are not comfortable about anything we will make sure that we will listen to your concerns and we will stop visiting you if you wish it.

**Benefits**

You and your baby will not receive any direct benefit for taking part in this study. However, you will receive intensive support for exclusive breastfeeding, monitoring of growth in the baby, examination and advice for treatment of illnesses. Also if you have any questions about your health we will be happy to answer them. We will pay for any test that we perform ourselves but we wont be able to pay for any other extra tests. We will not be able to pay for additional hospital investigations or treatment. The results of the study will help us answer important research questions about growth in babies under six months.

We will give you a small token of appreciation for your time (*site specific: Bangladesh, India, Pakistan, Tanzania, Nigeria sites*).

**Confidentiality**

We will not be sharing the identity of those participating in the research with people who are not part of the research team. Information about you and your baby that will be collected during the research will be stored safely and no one but the researchers will be able to see it. The information about you and your baby will have a number on it instead of your names. Only the researchers will know what your number is and we will keep that information under lock and key. It will not be shared with or given to anyone except the World Health Organization and the Data Safety Monitoring Board (which is a committee of experts aimed to ensure that the study is done well and the study participants are safe). We will keep your and your baby’s data permanently but all information about you will be anonymous – it will not contain your name or any personal identifying information.

**Sharing the results**

The knowledge that we get from doing this research will be shared with you before it is made widely available to the public. We will share the findings with you through meetings with your doctors and community meetings where ever possible. After these meetings, we will publish the results in order that other interested people may learn from the research. The publications will not identify any of the study participants.

**Right to refuse or withdraw**

You do not have to take part in this research if you do not wish to do so. You may also stop participating in the research at any time you choose without giving us any reason for the same.

**Who to contact**

If you have any questions you may ask them now or later, even after the study has started. If you wish to ask questions later, you may contact *[site specific details, name of PI, address, phone number, email address].*

This proposal has been reviewed and approved by the Ethics Committee of *[site specific details]*, which is a committee whose task it is to make sure that research participants are protected from harm. If you wish to find more about the ethics committee you can contact *[site specific details]*. It has also been reviewed by the Ethics Review Committee of the World Health Organization (WHO), which is funding and supporting the study.

You can ask me any more questions about any part of the research study, if you wish to. Do you have any questions?

**END OF MAIN STUDY INFORMATION SHEET**

**Nutritional management of growth faltering in infants aged under six months in Asia and Africa. An individually randomised trial. Informed consent form. Version 1.4. 8July2024**

**INFORMED CONSENT FORM FOR WOMEN AND INFANTS IN MAIN STUDY**

I have read the foregoing information, or it has been read to me. I have had the opportunity to ask questions about it and any questions that I have asked have been answered to my satisfaction. I consent voluntarily to participate as a participant in this research.

**Print name of participant_______________________________­­­­­­­­_**

**Signature of participant ___________________________**

**OR Thumb print of**

**participant**

**Date ___________________________**

**Day/month/year**

**If illiterate**

I have witnessed the accurate reading of the consent form to the potential participant, and the individual has had the opportunity to ask questions. I confirm that the individual has given consent freely.

**Print name of witne*ss*** __________________________

**Signature of witness_____________________________**

**Date ________ ___________________**

**Day/month/year**

**Statement by the researcher/person taking consent**

I have accurately read out the information sheet to the potential participant, and to the best of my ability made sure that the participant understands that the following will be done:

1. Her baby will be placed in one of the two groups as if by lottery which is similar to tossing a coin. Depending on which group the baby is in, he/she will either receive intensive breastfeeding support plus nutritional supplementation or intensive breastfeeding support alone without nutritional supplementation.
2. She will be asked some questions about her family and living conditions
3. Between day seven and fourteen after birth her baby’s weight, length, and head circumference will be measured
4. We will visit her home regularly to measure her baby’s weight and to ask about illnesses and feeding until the baby reaches six months.
5. She will be offered support from breastfeeding experts, nurses and doctors
6. She will be offered a nutritional milk supplement if the baby meets the study growth faltering criteria, and she is in the nutritional supplementation group.
7. At six months of age her baby’s weight, length, head, and upper arm circumference will be measured

I confirm that the participant was given an opportunity to ask questions about the study, and all the questions asked by the participant have been answered correctly and to the best of my ability. I confirm that the individual has not been coerced into giving consent, and the consent has been given freely and voluntarily.  A copy of the information sheet has been provided to the participant.

**Print name of researcher/person taking the consent________________________________**

**Signature of researcher /person taking the consent__________________________**

**Date __________________**

**Day/month/year**

**Signed copies of this consent form must be: 1) given to the woman and 2) placed in the infant’s research record.**

**END OF MAIN STUDY CONSENT FORM**

**Appendix 3. Spirit check list**

**SPIRIT Checklist for *Trials***


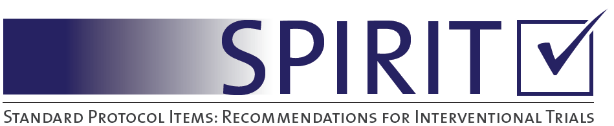
SPIRIT 2013 Checklist: Recommended items to address in a clinical trial protocol and related documents*

|  |  | **Reporting Item** | **Page number** | **Reason if not applicable** |
| --- | --- | --- | --- | --- |
| **Administrative information** | | | | |
| Title | [#1](https://www.goodreports.org/reporting-checklists/spirit/info/#1) | Descriptive title identifying the study design, population, interventions, and, if applicable, trial acronym | 1 |  |
| Trial registration | [#2a](https://www.goodreports.org/reporting-checklists/spirit/info/#2a) | Trial identifier and registry name. If not yet registered, name of intended registry | 3, 19 |  |
| Trial registration: data set | [#2b](https://www.goodreports.org/reporting-checklists/spirit/info/#2b) | All items from the World Health Organization Trial Registration Data Set | 19 |  |
| Protocol version | [#3](https://www.goodreports.org/reporting-checklists/spirit/info/#3) | Date and version identifier | 19 |  |
| Funding | [#4](https://www.goodreports.org/reporting-checklists/spirit/info/#4) | Sources and types of financial, material, and other support | 3,19 |  |
| Roles and responsibilities: contributorship | [#5a](https://www.goodreports.org/reporting-checklists/spirit/info/#5a) | Names, affiliations, and roles of protocol contributors | 17,18 |  |
| Roles and responsibilities: sponsor contact information | [#5b](https://www.goodreports.org/reporting-checklists/spirit/info/#5b) | Name and contact information for the trial sponsor | 18 |  |
| Roles and responsibilities: sponsor and funder | [#5c](https://www.goodreports.org/reporting-checklists/spirit/info/#5c) | Role of study sponsor and funders, if any, in study design; collection, management, analysis, and interpretation of data; writing of the report; and the decision to submit the report for publication, including whether they will have ultimate authority over any of these activities | 5,18 |  |
| Roles and responsibilities: committees | [#5d](https://www.goodreports.org/reporting-checklists/spirit/info/#5d) | Composition, roles, and responsibilities of the coordinating centre, steering committee, endpoint adjudication committee, data management team, and other individuals or groups overseeing the trial, if applicable (see Item 21a for data monitoring committee) | 5,18 |  |
| **Introduction** |  |  |  |  |
| Background and rationale | [#6a](https://www.goodreports.org/reporting-checklists/spirit/info/#6a) | Description of research question and justification for undertaking the trial, including summary of relevant studies (published and unpublished) examining benefits and harms for each intervention | 4 |  |
| Background and rationale: choice of comparators | [#6b](https://www.goodreports.org/reporting-checklists/spirit/info/#6b) | Explanation for choice of comparators | 4 |  |
| Objectives | [#7](https://www.goodreports.org/reporting-checklists/spirit/info/#7) | Specific objectives or hypotheses | 4,5 |  |
| Trial design | [#8](https://www.goodreports.org/reporting-checklists/spirit/info/#8) | Description of trial design including type of trial (eg, parallel group, crossover, factorial, single group), allocation ratio, and framework (eg, superiority, equivalence, non-inferiority, exploratory) | 5 |  |
| **Methods: Participants, interventions, and outcomes** | | | | |
| Study setting | [#9](https://www.goodreports.org/reporting-checklists/spirit/info/#9) | Description of study settings (eg, community clinic, academic hospital) and list of countries where data will be collected. Reference to where list of study sites can be obtained | 6 |  |
| Eligibility criteria | [#10](https://www.goodreports.org/reporting-checklists/spirit/info/#10) | Inclusion and exclusion criteria for participants. If applicable, eligibility criteria for study centres and individuals who will perform the interventions (eg, surgeons, psychotherapists) | 6 |  |
| Interventions: description | [#11a](https://www.goodreports.org/reporting-checklists/spirit/info/#11a) | Interventions for each group with sufficient detail to allow replication, including how and when they will be administered | 6-11 |  |
| Interventions: modifications | [#11b](https://www.goodreports.org/reporting-checklists/spirit/info/#11b) | Criteria for discontinuing or modifying allocated interventions for a given trial participant (eg, drug dose change in response to harms, participant request, or improving / worsening disease) | 11 |  |
| Interventions: adherance | [#11c](https://www.goodreports.org/reporting-checklists/spirit/info/#11c) | Strategies to improve adherence to intervention protocols, and any procedures for monitoring adherence (eg, drug tablet return; laboratory tests) | 11 |  |
| Interventions: concomitant care | [#11d](https://www.goodreports.org/reporting-checklists/spirit/info/#11d) | Relevant concomitant care and interventions that are permitted or prohibited during the trial | 11 |  |
| Outcomes | [#12](https://www.goodreports.org/reporting-checklists/spirit/info/#12) | Primary, secondary, and other outcomes, including the specific measurement variable (eg, systolic blood pressure), analysis metric (eg, change from baseline, final value, time to event), method of aggregation (eg, median, proportion), and time point for each outcome. Explanation of the clinical relevance of chosen efficacy and harm outcomes is strongly recommended | 5, Table 1 |  |
| Participant timeline | [#13](https://www.goodreports.org/reporting-checklists/spirit/info/#13) | Time schedule of enrolment, interventions (including any run-ins and washouts), assessments, and visits for participants. A schematic diagram is highly recommended (see Figure) | Figure 2 |  |
| Sample size | [#14](https://www.goodreports.org/reporting-checklists/spirit/info/#14) | Estimated number of participants needed to achieve study objectives and how it was determined, including clinical and statistical assumptions supporting any sample size calculations | 12 |  |
| Recruitment | [#15](https://www.goodreports.org/reporting-checklists/spirit/info/#15) | Strategies for achieving adequate participant enrolment to reach target sample size | 6,8 |  |
| **Methods: Assignment of interventions (for controlled trials)** | | | | |
| Allocation: sequence generation | [#16a](https://www.goodreports.org/reporting-checklists/spirit/info/#16a) | Method of generating the allocation sequence (eg, computer-generated random numbers), and list of any factors for stratification. To reduce predictability of a random sequence, details of any planned restriction (eg, blocking) should be provided in a separate document that is unavailable to those who enrol participants or assign interventions | 6,7 |  |
| Allocation concealment mechanism | [#16b](https://www.goodreports.org/reporting-checklists/spirit/info/#16b) | Mechanism of implementing the allocation sequence (eg, central telephone; sequentially numbered, opaque, sealed envelopes), describing any steps to conceal the sequence until interventions are assigned | 6,7 |  |
| Allocation: implementation | [#16c](https://www.goodreports.org/reporting-checklists/spirit/info/#16c) | Who will generate the allocation sequence, who will enrol participants, and who will assign participants to interventions | 6,7 |  |
| Blinding (masking) | [#17a](https://www.goodreports.org/reporting-checklists/spirit/info/#17a) | Who will be blinded after assignment to interventions (eg, trial participants, care providers, outcome assessors, data analysts), and how | p6,7 |  |
| Blinding (masking): emergency unblinding | [#17b](https://www.goodreports.org/reporting-checklists/spirit/info/#17b) | If blinded, circumstances under which unblinding is permissible, and procedure for revealing a participant’s allocated intervention during the trial | p6,7 |  |
| **Methods: Data collection, management, and analysis** | | | | |
| Data collection plan | [#18a](https://www.goodreports.org/reporting-checklists/spirit/info/#18a) | Plans for assessment and collection of outcome, baseline, and other trial data, including any related processes to promote data quality (eg, duplicate measurements, training of assessors) and a description of study instruments (eg, questionnaires, laboratory tests) along with their reliability and validity, if known. Reference to where data collection forms can be found, if not in the protocol | p7,8. Appendix 1 |  |
| Data collection plan: retention | [#18b](https://www.goodreports.org/reporting-checklists/spirit/info/#18b) | Plans to promote participant retention and complete follow-up, including list of any outcome data to be collected for participants who discontinue or deviate from intervention protocols | p7,8, Table 1 |  |
| Data management | [#19](https://www.goodreports.org/reporting-checklists/spirit/info/#19) | Plans for data entry, coding, security, and storage, including any related processes to promote data quality (eg, double data entry; range checks for data values). Reference to where details of data management procedures can be found, if not in the protocol | p12,13 |  |
| Statistics: outcomes | [#20a](https://www.goodreports.org/reporting-checklists/spirit/info/#20a) | Statistical methods for analysing primary and secondary outcomes. Reference to where other details of the statistical analysis plan can be found, if not in the protocol | p12-14, Appendix 4 |  |
| Statistics: additional analyses | [#20b](https://www.goodreports.org/reporting-checklists/spirit/info/#20b) | Methods for any additional analyses (eg, subgroup and adjusted analyses) | p13, Appendix 4 |  |
| Statistics: analysis population and missing data | [#20c](https://www.goodreports.org/reporting-checklists/spirit/info/#20c) | Definition of analysis population relating to protocol non-adherence (eg, as randomised analysis), and any statistical methods to handle missing data (eg, multiple imputation) | P14, Appendix 4 |  |
| **Methods: Monitoring** | | | | |
| Data monitoring: formal committee | [#21a](https://www.goodreports.org/reporting-checklists/spirit/info/#21a) | Composition of data monitoring committee (DMC); summary of its role and reporting structure; statement of whether it is independent from the sponsor and competing interests; and reference to where further details about its charter can be found, if not in the protocol. Alternatively, an explanation of why a DMC is not needed | p5,6 |  |
| Data monitoring: interim analysis | [#21b](https://www.goodreports.org/reporting-checklists/spirit/info/#21b) | Description of any interim analyses and stopping guidelines, including who will have access to these interim results and make the final decision to terminate the trial | p5, Appendix 4 |  |
| Harms | [#22](https://www.goodreports.org/reporting-checklists/spirit/info/#22) | Plans for collecting, assessing, reporting, and managing solicited and spontaneously reported adverse events and other unintended effects of trial interventions or trial conduct | p11,12 |  |
| Auditing | [#23](https://www.goodreports.org/reporting-checklists/spirit/info/#23) | Frequency and procedures for auditing trial conduct, if any, and whether the process will be independent from investigators and the sponsor | p15 |  |
| **Ethics and dissemination** | | | | |
| Research ethics approval | [#24](https://www.goodreports.org/reporting-checklists/spirit/info/#24) | Plans for seeking research ethics committee / institutional review board (REC / IRB) approval | p14,15 |  |
| Protocol amendments | [#25](https://www.goodreports.org/reporting-checklists/spirit/info/#25) | Plans for communicating important protocol modifications (eg, changes to eligibility criteria, outcomes, analyses) to relevant parties (eg, investigators, REC / IRBs, trial participants, trial registries, journals, regulators) | p14 |  |
| Consent or assent | [#26a](https://www.goodreports.org/reporting-checklists/spirit/info/#26a) | Who will obtain informed consent or assent from potential trial participants or authorised surrogates, and how (see Item 32) | p14,15 |  |
| Consent or assent: ancillary studies | [#26b](https://www.goodreports.org/reporting-checklists/spirit/info/#26b) | Additional consent provisions for collection and use of participant data and biological specimens in ancillary studies, if applicable | Not applicable | No current plans for ancillary studies |
| Confidentiality | [#27](https://www.goodreports.org/reporting-checklists/spirit/info/#27) | How personal information about potential and enrolled participants will be collected, shared, and maintained in order to protect confidentiality before, during, and after the trial | p14,15 |  |
| Declaration of interests | [#28](https://www.goodreports.org/reporting-checklists/spirit/info/#28) | Financial and other competing interests for principal investigators for the overall trial and each study site | p19 |  |
| Data access | [#29](https://www.goodreports.org/reporting-checklists/spirit/info/#29) | Statement of who will have access to the final trial dataset, and disclosure of contractual agreements that limit such access for investigators | p12,18,19 |  |
| Ancillary and post trial care | [#30](https://www.goodreports.org/reporting-checklists/spirit/info/#30) | Provisions, if any, for ancillary and post-trial care, and for compensation to those who suffer harm from trial participation | p15 |  |
| Dissemination policy: trial results | [#31a](https://www.goodreports.org/reporting-checklists/spirit/info/#31a) | Plans for investigators and sponsor to communicate trial results to participants, healthcare professionals, the public, and other relevant groups (eg, via publication, reporting in results databases, or other data sharing arrangements), including any publication restrictions | p14,16 |  |
| Dissemination policy: authorship | [#31b](https://www.goodreports.org/reporting-checklists/spirit/info/#31b) | Authorship eligibility guidelines and any intended use of professional writers | p17,18 |  |
| Dissemination policy: reproducible research | [#31c](https://www.goodreports.org/reporting-checklists/spirit/info/#31c) | Plans, if any, for granting public access to the full protocol, participant-level dataset, and statistical code | p19 |  |
| **Appendices** | | | | |
| Informed consent materials | [#32](https://www.goodreports.org/reporting-checklists/spirit/info/#32) | Model consent form and other related documentation given to participants and authorised surrogates | Appendix 2 |  |
| Biological specimens | [#33](https://www.goodreports.org/reporting-checklists/spirit/info/#33) | Plans for collection, laboratory evaluation, and storage of biological specimens for genetic or molecular analysis in the current trial and for future use in ancillary studies, if applicable | Not applicable | No plans for biological specimens |

This checklist was read in conjunction with the SPIRIT 2013 Explanation & Elaboration for important clarification on the items. Amendments to the protocol should be tracked and dated. The SPIRIT checklist is copyrighted by the SPIRIT Group under the Creative Commons “[Attribution-NonCommercial-NoDerivs 3.0 Unported](http://www.creativecommons.org/licenses/by-nc-nd/3.0/)” license. This checklist can be completed online using https://www.goodreports.org/, a tool made by the EQUATOR Network in collaboration with Penelope.ai

Ref: Chan A-W, Tetzlaff JM, Gøtzsche PC, Altman DG, Mann H, Berlin J, Dickersin K, Hróbjartsson A, Schulz KF, Parulekar WR, Krleža-Jerić K, Laupacis A, Moher D. SPIRIT 2013 Explanation and Elaboration: Guidance for protocols of clinical trials. BMJ. 2013;346:e7586

**Appendix 4. Statistical analysis plan**

**Please see separate file**

**Appendix 5. Ethical approval**

Please see separate file

**Appendix 6. Funding information**

Please see separate file
