# Supplementary material for: Nutritional management of growth faltering in infants aged under six months in Asia and Africa: study protocol for a multicentre randomised trial (BRANCH, BReAstfeediNg Counselling and management of growtH)
Source: Trials. 2025 Nov 6;26:474. doi: 10.1186/s13063-025-09034-y (PMC12590774; doi:10.1186/s13063-025-09034-y)
Supplement: Supplementary file 4 — Additional file 4: Appendix 4. Statistical analysis plan [file 13063_2025_9034_MOESM4_ESM.docx]

**Statistical Analysis Plan**

**Nutritional management of growth faltering in infants aged under six months in Asia and Africa: an individually-randomised controlled trial (BRANCH, Breastfeeding counselling and management of growth)**

| **Version Number / Status (Draft/Final/Amendment)** | 1.0/Final |
| --- | --- |
| **Date** | 21 Aug 2024 |
| **Protocol Number / Date** | 1.4 / 8^th^ July 2024 |
| **Standard Operating Procedures** | 1.3 / 2^nd^ Aug 2024 |
| **Trial registration number** | ACTRN12624000704594 |

**Revision History**

| **Version** | **Date** | **Revision description** |
| --- | --- | --- |
|  |  |  |
|  |  |  |
|  |  |  |
|  |  |  |
|  |  |  |

**Authors**

| **Name** | **Signature** | **Date** |
| --- | --- | --- |
| Lisa Hurt  Coordinator of trial statistics team | **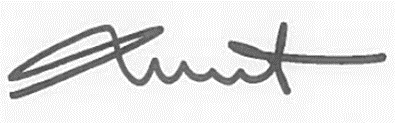** | 21 August 2024 |
| Rhian Daniel  Senior statistician | **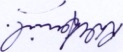** | 21 August 2024 |
| Karen Edmond  Coordinator of the trial | 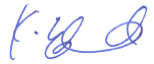 | 21 August 2024 |

**Table of Contents**

[1. INTRODUCTION 7](#_Toc170375384)

[2. BACKGROUND 7](#_Toc170375385)

[2.1 Rationale 7](#_Toc170375386)

[2.2 Aims and objectives 7](#_Toc170375387)

[2.2.1 Aim 7](#_Toc170375388)

[2.2.2 Primary objective 7](#_Toc170375389)

[2.2.3 Secondary objectives 7](#_Toc170375390)

[3. TRIAL DESIGN 8](#_Toc170375391)

[3.1 Eligibility 8](#_Toc170375392)

[3.1.1 Inclusion and exclusion criteria 8](#_Toc170375393)

[3.1.2 Eligibility criteria for surveillance of women of reproductive age 8](#_Toc170375394)

[3.1.3 Eligibility criteria for pregnancy surveillance 9](#_Toc170375395)

[3.1.4 Eligibility criteria for enrolment 9](#_Toc170375396)

[3.1.5 Eligibility criteria for interventions 9](#_Toc170375397)

[3.3 Definitions of outcomes 10](#_Toc170375398)

[3.3.1 Date conventions for outcome definitions 10](#_Toc170375399)

[3.3.2 Definition of primary outcome 11](#_Toc170375400)

[3.3.3 Definition of secondary outcomes 11](#_Toc170375401)

[3.4 Sample size calculation 13](#_Toc170375402)

[3.5 Study procedures 13](#_Toc170375403)

[3.5.1 Randomization 13](#_Toc170375404)

[3.5.2 Allocation concealment 14](#_Toc170375405)

[3.5.3 Blinding 14](#_Toc170375406)

[4. DATA COLLECTION, PROCESSING AND MONITORING 14](#_Toc170375407)

[4.1 Surveillance of women of reproductive age 14](#_Toc170375408)

[4.2 Surveillance of pregnant women 15](#_Toc170375409)

[4.3 Postpartum surveillance of mother and infant(s) 15](#_Toc170375410)

[4.4 Data management and transfer from sites 16](#_Toc170375411)

[4.5 Data management and monitoring 16](#_Toc170375412)

[5. STATISTICAL METHODS 16](#_Toc170375413)

[5.1 Descriptive statistics 16](#_Toc170375414)

[5.1.1 Description of the population 16](#_Toc170375415)

[5.1.2 Participant flow 17](#_Toc170375416)

[5.1.3 Baseline comparability of randomized groups 17](#_Toc170375417)

[5.2 Comparative analyses 17](#_Toc170375418)

[5.2.1 Safety analyses 17](#_Toc170375419)

[5.2.2 Analysis of primary outcome 18](#_Toc170375420)

[5.2.3 Analysis of secondary outcomes 18](#_Toc170375421)

[5.3 Missing data 19](#_Toc170375422)

[5.4 Additional analyses 19](#_Toc170375423)

[5.4.1 Sub-group analyses 19](#_Toc170375424)

[5.4.2 Sensitivity analysis 19](#_Toc170375425)

[6. CHANGES TO PLANNED ANALYSES 20](#_Toc170375426)

[7. INTERIM ANALYSES 20](#_Toc170375427)

[8. REFERENCES 22](#_Toc170375428)

[APPENDICES 22](#_Toc170375429)

[Appendix 1: Population description 23](#_Toc170375430)

[Appendix 2: Trial flow diagram 25](#_Toc170375431)

[Appendix 3: Participant characteristics 26](#_Toc170375432)

[Appendix 4: Serious adverse events 29](#_Toc170375433)

[Appendix 5: Analysis of primary outcome 30](#_Toc170375434)

[Appendix 6: Analyses of secondary outcomes 31](#_Toc170375435)

**List of abbreviations**

| AGA | Appropriate for gestational age |
| --- | --- |
| DSMB | Data Safety Monitoring Board |
| LBW | Low birthweight |
| MUAC | Mid upper arm circumference |
| SD | Standard deviation |
| SGA | Small for gestational age |
| SOP | Standard Operating Procedures |
| WHO | World Health Organization |

# **1. INTRODUCTION**

This document provides full details of the statistical analysis plan for the BRANCH trial, including shell figures and tables. It has been drafted in accordance with guidelines for the content of statistical analysis plans in clinical trials (Gamble et al 2017). It should be read in conjunction with the Trial Protocol (version 1.4, 8 July 2024) and Standard Operating Procedures (SOPs, version 1.3, 2 August 2024), which include a full description of the trial definitions and methods.

# **2. BACKGROUND**

## **2.1 Rationale**

There is a lack of evidence-based guidelines for management of growth faltering in the first 6 months of life. Evaluation of interventions to manage growth faltering in the first six months after birth was one of the highest priority research questions identified during an informal World Health Organization (WHO) consultation in January 2019.

## **2.2 Aims and objectives**

### 2.2.1 Aim

The overall aim of this trial is to determine the effect of intensive breastfeeding counselling and support plus nutritional supplementation compared with intensive breastfeeding counselling and support alone on mortality, morbidity and growth in infants aged 0-6 months in seven low resource settings in South Asia and Sub-Saharan Africa.

### 2.2.2 Primary objective

The primary objective is to determine the effect of intensive breastfeeding counselling and support plus nutritional supplementation on wasting-free survival at 6 completed months of age.

### 2.2.3 Secondary objectives

Secondary objectives are:

- To determine the effect of intensive breastfeeding counselling and support plus nutritional supplementation on common childhood morbidities, underweight, wasting and stunting outcomes.
- To determine the effect of intensive breastfeeding counselling and support plus nutritional supplementation on the primary outcome in subgroups based on birth weight and gestational age at birth (term appropriate for gestational age [AGA], preterm AGA, term small for gestational age [SGA], preterm SGA), infant sex, study site, and growth faltering.
- To optimize breastfeeding support for vulnerable newborns and infants with growth faltering in low resource settings in South Asia and Sub-Saharan Africa.
- To develop operational guidance on breastfeeding and intensive lactation support that can be used across low and middle income countries.

# **3. TRIAL DESIGN**

This is a multi-centre, parallel group, individually-randomized, non-blinded, controlled study implemented in seven countries: three in Asia (Bangladesh, India and Pakistan) and four in Africa (Ethiopia, Nigeria, Tanzania and Uganda).

## **3.1 Eligibility**

### 3.1.1 Inclusion and exclusion criteria

All women of reproductive age in the study areas will be identified at the start of the study. Consent procedures will vary by site (as set out in the Protocol) according to local requirements relating to legal guardians. If women consent to enter surveillance, they will be contacted at least every 12 weeks to identify possible pregnancies. All women who are confirmed by ultrasound to be less than 20 weeks pregnant and who consent to pregnancy surveillance will be followed-up monthly until 32 weeks, then weekly until the infant is born. Enrolment will occur when the infant is between 7 and 13 days of age (inclusive) if they fulfil the inclusion criteria below.

### 3.1.2 Eligibility criteria for surveillance of women of reproductive age

- Women 18-49 years who are married – Bangladesh, Ethiopia, India, Tanzania
- Women 15-49 years who are married – Pakistan
- Women 15-49 years regardless of marital status – Nigeria, Uganda

### 3.1.3 Eligibility criteria for pregnancy surveillance

- Viable pregnancy is confirmed by study team at ultrasound
- Gestational age is less than 20 weeks by study team at ultrasound
- Informed consent obtained as specified in Trial Protocol

### 3.1.4 Eligibility criteria for enrolment

Mother-infant dyads will be randomized, with the following inclusion criteria:

- Ultrasound-based gestational age before 20 weeks of gestation is available
- Infant was born at 28 weeks gestation or more
- Birth weight taken within 72 hours after birth
- Infant has been fed breastmilk at any time since birth
- The infant is > = 7 and < 14 days of age
- Singleton or twin births
- Informed consent obtained as specified in Trial Protocol

Exclusion criteria:

- The biological mother has died before eligibility screening
- Infant has a major abnormality or condition which is impairing feeding or nutrition (such as severe cleft palate, severe asphyxia, severe respiratory distress syndrome, need for mechanical ventilation, severe necrotising enterocolitis, major surgery)
- Family does not intend to live in the study area for six months
- The mother has given birth to three or more infants (that is, twins are included but triplets or quadruplets etc are excluded)
- An infant has already been recruited and is under surveillance from the same household (that is, the infant is currently under active follow-up by the study team).

### 3.1.5 Eligibility criteria for interventions

All terms used below (slow weight gain; growth concern; growth faltering; intensive breastfeeding counselling and support; and nutritional supplementation) are defined in the Trial Protocol.

- All mothers will receive breastfeeding promotion.
- All mothers of infants identified as having **slow weight gain** or **growth concern** will receive intensive breastfeeding counselling and support.
- Infants identified as **growth faltering** will either receive intensive breastfeeding counselling and support plus nutritional supplementation or intensive breastfeeding counselling and support plus usual care, depending on randomization.
- Infants identified to have **growth concern** for the first time at week 22 or later will receive nutritional supplementation (if randomized to that group) as soon as possible (and within a maximum of 7 days) without waiting for the growth assessment at 6 months.

## **3.3 Definitions of outcomes**

### 3.3.1 Date conventions for outcome definitions

Table 1 displays the time period definitions used in the trial. We will use hours to define the first week, then days for the remainder of the trial.

| **Table 1** |  |
| --- | --- |
| **Time period** | **Definition** |
| Day 0 | 0 to 23.99 hours (i.e. 0 to < 24 hours) |
| Day 1 | 24 to 47.99 hours (i.e. 24 to < 48 hours) |
| Day 2 | 48 to 71.99 hours (i.e. 48 to < 72 hours) |
| Day 3 | 72 to 95.99 hours (i.e. 72 to < 96 hours) |
| Day 4 | 96 to 119.99 hours (i.e. 96 to < 120 hours) |
| Day 5 | 120 to 143.99 hours (i.e. 120 to < 144 hours) |
| Day 6 | 144 to 167.99 hours (i.e. 144 to < 168 hours) |
| Week 1 | Day 0 to 6, 0 to 167.99 hours, 0 to < 168 hours |
| Week 2 | Day 7 to 13 |
| 6 completed months | 182-209 days (inclusive of both days) |
| Every 2 weeks | Every 14 days |

### 3.3.2 Definition of primary outcome

The primary outcome in the trial is a composite outcome, defined as wasting-free survival at 6 completed months of age. It will be expressed per 1000 enrolled infants.

***Numerator***

The numerator will be wasting-free survival (a composite of wasting and deaths) at 6 completed months. 6 completed months will be defined as 182 plus 27 days (to 209 days inclusive), and as close to 182 days as possible. The assessment time point for both wasting and deaths being on the same day unless a death has been reported at an earlier time point.

Wasting will be defined as weight for length z-score lower than −2 standard deviations (SD) from the median of the WHO Growth Standards, measured between days 182 and 209 days (WHO 2024).

Deaths will be defined as deaths from all causes between enrolment and 6 completed months of age. The assessment time point for deaths will be on the same day as wasting unless a death has been reported at an earlier time point.

To be included in the primary analysis, infants in the nutritional supplementation group must have started receiving the supplement before 182 days (to day 181 inclusive).

***Denominator***

The denominator for the analysis will be all randomized infants with known vital status and measurement data at 6 completed months of age.

Vital status will be ascertained at 6 completed months, that is, 182 plus 27 days (to 209 days inclusive), and as close to 182 days as possible.

### 3.3.3 Definition of secondary outcomes

Secondary outcomes are:

- All-cause mortality from enrolment to 6 completed months of age (that is, to day 209 inclusive)
- Wasting (weight for length z-score < −2 SD) at 6 completed months
- Severe wasting (weight for length z-score < −3 SD) at 6 completed months
- Underweight (weight-for-age z-score < -2 SD) at 6 completed months
- Stunting (length-for-age z-score < −2 SD) at 6 completed months
- Concurrent wasting and stunting (composite measure of wasting and stunting) at 6 completed months
- Weight-for-age z-score at 6 completed months
- Length-for-age z-score at 6 completed months
- Weight-for-length z-score at 6 completed months
- At least one hospitalisation^*^ for any cause from enrolment to 6 completed months of age
- At least one hospitalisation for diarrhoea from enrolment to 6 completed months of age
- At least one hospitalisation for pneumonia from enrolment to 6 completed months of age
- At least one hospitalisation for other infections from enrolment to 6 completed months of age
- At least one hospitalisation for all other causes from enrolment to 6 completed months of age
- Any care-seeking^**^ from enrolment to 6 completed months of age
- Breastfeeding practice in the past 24 hours, at 5 completed months of age^†^

* A hospitalisation is defined as a primary caregiver report of either an inpatient admission (where registration number is allotted) or a stay of more than or equal to 24 consecutive hours in treatment facility / hospital (excluding waiting time). This includes treatment in the emergency services, diarrhoea management room, or any paediatric ward in the institution.

** Care seeking is defined as at least one primary caregiver report of health care-seeking, including any face-to-face visit to any health facility, pharmacist or drug-seller

^†^ Breastfeeding practice in the past 24 hours will be defined as breastfed or not breastfed. This will be measured at 5 completed months. Data collected at 154 to 181 days (and as close to 154 days as possible) will be used for this outcome because feeding practices are known to change at around 6 months of age in accordance with international guidance (for example, with the introduction of complementary solid foods).

## **3.4 Sample size calculation**

The following assumptions have been used for sample size calculations:

- In the comparison group, mortality between enrolment and 6 months of age will be 1-2% and wasting at 6 months among survivors will be 6-8%. The primary outcome will therefore be 9% (range 7% to 10%) in the comparison group.
- The hypothesized primary outcome in the intervention group will be 20% lower compared to the comparison group, i.e. 7.2% infants (range 5.6% to 8.0%).
- 95% confidence level, 90% power.
- 10% loss to follow up during the study period.

With the above assumptions, the total sample required is 11,000 infants. This would also allow for the detection of a 20% reduction in the primary outcome if this had a prevalence of 7% in the control group with 80% power at the 95% significant level.

Simulations that account for the complex analysis methods required to adjust for multiple births and the stratified randomization described below (using generalized estimating equations) suggest that the power of the study remains at 87%.

The assumptions used in the sample size calculations will be monitored in the data during the regular monitoring by the project team and by the Data Safety Monitoring Board (in accordance with their terms of reference).

## **3.5 Study procedures**

### 3.5.1 Randomization

Eligible mother-infant dyads will be randomized in a 1:1 ratio to the intensive breastfeeding counselling and support plus nutritional supplementation or intensive breastfeeding counselling and support groups. The randomization list will be prepared by an independent statistician using blocks of size 10.

Each site will have a separate randomization list, with additional stratification into eight groups according to the characteristics of her infant(s): singleton term appropriate for gestational age (AGA); singleton term SGA; singleton preterm AGA; singleton preterm SGA; multiple term AGA; multiple term SGA; multiple preterm AGA; and multiple preterm SGA. Both twins from a multiple pregnancy will be in the same trial group for ethical and logistical reasons. If there is discordance with regard to the AGA or SGA status of the twins, the SGA randomization list will be used.

### 3.5.2 Allocation concealment

Allocation to intervention and control groups will be done through a server-based system, and the recruiters will not be aware of the next allocation when recruiting infants. Allocation will happen when the mothers-infant dyads are randomized (when the infant is aged between 7 and 13 days inclusive) and stored within the mother’s database record, with the information only accessed if the infant is identified as having growth faltering at a later point in the trial.

### 3.5.3 Blinding

Blinding of participants and data collection teams will not be possible because of the nature of interventions in this trial. However, attempts will be made to keep the independent outcome assessment team unaware of the group allocation as far as this is possible. The study statistics team will remain blinded throughout the trial and during the primary analysis of trial data.

# **4. DATA COLLECTION, PROCESSING AND MONITORING**

## **4.1 Surveillance of women of reproductive age**

Each site will maintain an up-to-date register of all eligible women of reproductive age in the study area who will participate in reproductive age surveillance. Each woman will be followed up at a minimum of every 12 weeks using telephone or face to face interviews to ask if the woman thinks she might be pregnant or has missed any periods (with the range in frequency to allow for minor differences in procedures by site, depending on whether they already have active demographic surveillance systems in place).

## **4.2 Surveillance of pregnant women**

If the woman thinks she is pregnant or has missed two consecutive periods, or has a positive pregnancy test, she will be offered a trans-abdominal ultrasound. When a pregnancy and gestational age is confirmed by ultrasound, the pregnant woman will be consented for pregnancy follow up. This will involve follow up in person or by telephone at a minimum of every 4 weeks until the eight month of pregnancy and then weekly until the infant is born.

## **4.3 Postpartum surveillance of mother and infant(s)**

The first follow-up of the mother and infant(s) will be within 72 hours of birth to collect basic data (live or stillborn; current status; and birth weight) on the infant(s). Where there is a multiple birth, data on both twins will be collected throughout all trial procedures.

On day 7 (and before day 14), consent will be taken for enrolment. Socio-demographic, obstetric (including mode of delivery and birth complications) and neonatal data will be collected at this time, and the infant’s weight, length, and head circumference will be measured.

Infant surveillance will take place every 7 days for infants under 4 weeks of age, and every 14 days for infants between the ages of 1 and 6 months. Data will be collected on: feeding practices (i.e. exclusive breastfeeding, other intake of fluids or foods), and health status (episodes of diarrhoea, respiratory infection, other infections, other health problems, and hospitalisations). Families will be asked how they have fed their infant(s) since the last visit and in the last 24 hours. The surveillance team will also ask about any illnesses and hospital visits that the infant has had since the last visit including runny stools (diarrhoea) and fast breathing (pneumonia). Infants will also have their weight measured using standardised weight scales.

At between 182 and 209 days inclusive, all of the above data will be collected plus length, head circumference and MUAC. The infant’s vital status on day 209 will also be collected, with this data collection occurring between 210 and 237 days.

## **4.4 Data management and transfer from sites**

Data will be collected electronically using tablets or hand-held devices using a bespoke data management system developed specifically for this trial. Range and consistency checks will be built in to ensure data quality. Real-time data will be transferred to local and web-based servers. Logic errors and checks across different forms will be run regularly and queries generated are given to study team for resolution and corrections incorporated.

## **4.5 Data management and monitoring**

Individual country datasets will be uploaded from each site every four weeks to the WHO coordinating team and the study statistics team at Cardiff University on share point via a file-transfer protocol developed for this study. Data monitoring for accrual and data accuracy and completeness will be conducted for each site, and for the pooled data, every four weeks. The detailed specifications for this will be set out in the Central Data Monitoring SOP.

# **5. STATISTICAL METHODS**

The primary analyses will be pooled across all sites.

Analyses will be on an ‘intention-to-treat’ basis, with infants analysed on the basis of the group to which the mother-infant dyad was randomized, irrespective of whether participants received the intensive breastfeeding counselling and support and nutritional supplementation (if randomized to this group) or the “dose” of either received.

## **5.1 Descriptive statistics**

### 5.1.1 Description of the population

To understand the characteristics of the population in which the study has been conducted, data on mortality, morbidity, growth, infant feeding patterns, and care-seeking (including treatments received for any growth concern) in the first six months of life will be summarized for the whole population and among infants whose mothers receive breastfeeding counselling and support (see **Appendix 1: Population description**).

### 5.1.2 Participant flow

The flow and number of infants through assessment of eligibility, randomization, completeness of follow-up, and analysis will be presented, along with reasons for exclusions and withdrawals (see **Appendix 2: Trial flow diagram**).

### 5.1.3 Baseline comparability of randomized groups

Baseline characteristics of infants and their mothers and families will be compared in the intervention and control groups to check if randomization has achieved adequate balance in these characteristics between the groups. Summary values (means, proportions) for infant, maternal and household characteristics in the intervention and control groups will be presented in the baseline table (see **Appendix 3: Participant characteristics tables**). We will not perform any significance tests.

## **5.2 Comparative analyses**

### 5.2.1 Safety analyses

The serious adverse events for the trial are:

- Mortality from any cause at any point in the first six months of life in an enrolled infant
- Hospitalisation from any cause at any point in the first six months of life in an enrolled infant
- Mortality or hospitalisation for any cause at any point in the first six months of life in an enrolled infant
- Mortality associated with vomiting or diarrhoea* at any point in the first six months of life in an enrolled infant
- Hospitalisation for vomiting or diarrhoea at any point in the first six months of life in an enrolled infant
- Mortality associated with or hospitalisation for vomiting or diarrhoea at any point in the first six months of life in an enrolled infant

* As noted on the serious adverse event form by the senior team member completing this form

Data will be presented as risk ratios with 95% confidence intervals as shown in **Appendix 4: Serious adverse events**, and analysed using the methods described below for the primary outcome.

### 5.2.2 Analysis of primary outcome

The primary analysis will assess the effect of the intervention compared with the comparison group on a binary outcome, wasting-free survival at 6 completed months of age (see **Appendix 5: Analysis of primary outcome**).

The intervention group will be compared against the control group for the primary outcome using unadjusted and adjusted risk ratios with 95% CIs. To account for the potential clustering introduced by including twin births and repeated pregnancies in the same woman,

generalized estimating equations with a logit link will be used for the adjusted analyses, with the mother included as a cluster and all of the other factors included in the stratified randomisation (site, AGA or SGA, term or preterm, singleton or multiple birth) included as covariates. The QIC (quasi-likelihood under the independence model criterion) will be used to choose between an independence, exchangeable and unstructured correlation structure (Cui 2007). The resulting estimated coefficients (on the log odds scale) will be converted to risk ratios via the predicted risks, and the uncertainty about the resulting risk ratios estimated using the delta method.

### 5.2.3 Analysis of secondary outcomes

The secondary analyses are to determine the effect of intervention on mortality, wasting, severe wasting, underweight, concurrent wasting and stunting, and morbidity, measured at 6 completed months of life, and breastfeeding practice measured at 5 completed months (see **Appendix 6: Analyses of secondary outcomes**). In addition, we will assess and report the proportion of infants in the intervention group who require a nutritional milk supplement.

The effect of interventions on binary secondary outcomes will be assessed using the same models as for primary outcomes.

For continuous outcomes, means and standard deviations will be presented for all z scores and morbidities. Generalized linear models of the Gaussian family with an identity-link function will be used to estimate the effect sizes (difference in means and 95% CIs).

## **5.3 Missing data**

The primary analysis will be a complete case analysis (including only infants with complete outcome data) if there is less than 5% missing data on the primary outcome, or if we are unable to identify predictors for the missingness. If a complete case analysis is not appropriate, we will account for the missing data using multiple imputation, with the missing data assumed to be missing at random.

## **5.4 Additional analyses**

### 5.4.1 Sub-group analyses

We will conduct subgroup analyses for the effects of the intervention:

1. by gestational age and birth weight in four groups: term AGA, preterm AGA, term SGA, preterm SGA;
2. by infant sex;
3. by study site (individually and whether the site is in Africa or Asia); and
4. in growth faltered and non-growth faltered infants separately.

The relative measures of effect within each of these subgroups will be estimated. We will conduct a test of homogeneity of effects across the subgroups and report a p value. Unless there is strong evidence against the null hypothesis of homogeneity of effects (i.e. p<0.001) the overall risk ratio will be considered as the most useful guide to the approximate relative risks in all subgroups.

### 5.4.2 Sensitivity analysis

The following sensitivity analyses have been pre-specified:

- Including only infants who have remained in the trial for at least 7 days after growth faltering has been identified;
- Including only infants who have remained in the trial for at least 14 days after growth faltering has been identified;
- Excluding critical protocol violations (as defined in the SOP);
- Using different definitions of infant mortality (infant mortality defined as mortality to 182 days only; and defined as mortality to the end of the follow up period of 209 days);
- Using different definitions of care seeking (allowing for a wider definition of healthcare providers in accordance with the different systems of care that exist in the sites);
- Additional adjustment (that is, in addition to the adjustment for stratification variables already specified above) if there are imbalances in other variables that may influence the primary outcome. Whether the variables are likely to influence the primary outcome and the level of imbalance which would trigger adjustment will be discussed with domain experts and the principal investigators at the time of the analyses, with all decisions reported clearly in any publications of the results.

# **6. CHANGES TO PLANNED ANALYSES**

None to date.

# **7. INTERIM ANALYSES**

The Data Monitoring Safety Board (DSMB) will review trial data in accordance with the schedule set out in their Terms of Reference (summarised in Table 2, next page).

The DSMB will:

1. Monitor data on recruitment and events (growth faltering and the primary outcome) within the trial (progress monitoring);
2. Monitor serious adverse events (safety monitoring);
3. Evaluate data on serious adverse events by trial group (safety analysis);
4. Evaluate interim analyses for benefits or harms of the intervention, to determine early differences in the primary outcome between trial groups (interim analyses).

| **Table 2** | | | | | |
| --- | --- | --- | --- | --- | --- |
|  |  | **Not by trial group** | | **By trial group** | |
| **Time point**^1 2^ | **Infants available for analysis**^3^ | **Progress monitoring** | **Safety monitoring** | **Safety analysis** | **Interim analysis** |
| Project month 3 | N~600  (~5% enrolled) | Yes | Yes | No | No |
| Project month 7 | N~2750  (25% enrolled) | Yes | Yes | Yes | No |
| Project month 10 | N~4800  (~44% enrolled) | Yes | Yes | No | No |
| Project month 14 | N~7200  (~65% enrolled)  N=4400  (40% with outcome) | Yes  No | Yes  No | Yes  Yes | No  Yes |
| Project month 17 | N~9000  (~82% enrolled) | Yes | Yes | No | No |
| Project month 21 | N=11000  (100% enrolled) | No | Yes | No | No |

^1^ Project months: Month 1 = month that the first baby is recruited; month 18 = month that the last baby is recruited; month 24 = month that the last baby is followed up to 6 months

^2^ The DSMB will meet online at months 7 and 14. At the other time points, the DSMB will decide whether to have an online meeting after reviewing the data.

^3^ Approximations based on expected recruitment of 600 infants per month. These also allow for 8 weeks of data processing after data are collected (4 weeks at the site including time to resolve field queries; 4 weeks of processing once uploaded to WHO).

The stopping rule for conducting an analysis of the primary outcome when 40% of infants have completed follow-up, along with alpha-spending implications of this, are summarised in Table 3.

| **Table 3** | |
| --- | --- |
| **Total number of infants with outcome** | **Alpha^1^** |
| 40% (N=4400) | 0.0007883 |
| End of study (N=9900)^2^ | 0.04921 |

^1^ Using O’Brien-Fleming and Lan and DeMets methods

^2^ Allowing for 10% loss to follow-up

# **8. REFERENCES**

Cui J. QIC program and model selection in GEE analyses. *The Stata Journal* 2007; 7(2):209-20.

Gamble C, Krishan A, Stocken D, Lewis S, Juszczak E, Doré C, Williamson PR, Altman DG, Montgomery A, Lim P, Berlin J, Senn S, Day S, Barbachano Y, Loder E. Guidelines for the content of statistical analysis plans in clinical trials. *JAMA* 2017; 318(23): 2337-43.

ICH Harmonised Tripartite Guideline. Statistical Principles for Clinical Trials. *Statistics in Medicine* 1999; 18: 1905-1942.

World Health Organization. Malnutrition in children. Geneva; World Health Organization. Available from www.who.int/data/nutrition/nlis/info/malnutrition-in-children (accessed 25/04/2024).

# **APPENDICES**

Appendix 1: Population description

Appendix 2: Trial flow diagram

Appendix 3: Participant characteristics tables

Appendix 4: Safety analyses

Appendix 5: Analysis of primary outcome

Appendix 6: Analyses of secondary outcomes

## **Appendix 1: Population description**

| **Characteristic** | | | | **All infants** | **Infants identified as having slow weight gain and growth concern** | **Infants identified as growth faltering** |
| --- | --- | --- | --- | --- | --- | --- |
| Number of mother-infant dyads randomised | | | |  |  |  |
| Number of infants | | | |  | N (%) of all infants recruited | N (%) of all infants recruited |
|  | | | Singleton infants  Infants from multiple births |  |  |  |
|  | | | Term Appropriate for Gestational Age  Term Small for Gestational Age  Preterm Appropriate for Gestational Age  Preterm Small for Gestational Age |  |  |  |
|  | | | Bangladesh  Ethiopia  India  Nigeria  Pakistan  Tanzania  Uganda |  |  |  |
|  | | | Post-enrolment infant mortality to six months per 1000 livebirths, N (%) of infants recruited |  |  |  |
|  | | | Wasting identified at 6 months, N (%) of infants recruited |  |  |  |
|  | | | Primary outcome of trial, N (%) of infants recruited |  |  |  |
| Number of infants with complete outcome data (that is, with complete information for anthropometry and vital status) | | | |  | N (%) of all infants with outcome data | N (%) of all infants with outcome data |
|  | | | Singleton infants  Infants from multiple births |  |  |  |
|  | | | Term Appropriate for Gestational Age  Term Small for Gestational Age  Preterm Appropriate for Gestational Age  Preterm Small for Gestational Age |  |  |  |
| Post-enrolment infant mortality to six months per 1000 livebirths, N (%) of infants with outcome data | | | |  |  |  |
| Wasting identified at 6 months, N (%) of infants with outcome data | | | |  |  |  |
| Primary outcome of trial, N (%) of infants with outcome data | | | |  |  |  |
| Severe morbidity* | | | |  |  |  |
|  | At least one all-cause hospitalisation, N (%)  Hospitalisation for diarrhoea, N (%)  Hospitalisation for pneumonia, N (%)  Hospitalisation for other infections, N (%)  Hospitalisation for other causes, N (%) | | |  |  |  |
| Nutrition at 6 completed months | | | |  |  |  |
|  | Wasting, N (%)  Severe wasting, N (%)  Underweight, N (%)  Stunting N, (%)  Concurrent wasting and stunting, N (%) | | |  |  |  |
| Breastfeeding | | | |  |  |  |
|  | | Exclusive breastfeeding at 1 month, N (%)  Exclusive breastfeeding at 3 months, N (%)  Exclusive breastfeeding at 5 months, N (%) | |  |  |  |
| Care-seeking* | | | |  |  |  |
|  | | At least one primary caregiver report of any health care-seeking, N (%) | |  |  |  |

* At least one episode during follow-up

## **Appendix 2: Trial flow diagram**

|  | | | Assessed for eligibility  (women, n = …; infants, n = …) | | | | |  | | | |
| --- | --- | --- | --- | --- | --- | --- | --- | --- | --- | --- | --- |
|  | |  | | |  | |  | | | | |
|  | |  | | |  | | Excluded (women, n = …; infants, n = …)  Not meeting inclusion criteria  (women, n = …; infants, n = …)   - Mother died (n = …) - Family did not intend to live in study area for 6 months (n = …) - Another infant was under surveillance in the household (n = …) - No USG data at <20 weeks gestation available (n = …) - Three or more infants born (n = …) - Infant born at <28 weeks (n = …) - Infant not >=7 and <14 days at recruitment visit (n = …) - Infant birthweight not measured within 72 hours (n = …) - Infant not fed breast milk at any time since birth (n = …) - Infant had major anomaly which impacted feeding or nutrition (n = …)   Refused to participate (women, n = …; infants, n = …)  Other reason (n = …) | | | | |
|  |  |  |  |  |  | |  |  |  |  |  |
|  | |  | | |  | |  | | | | |
|  | | | Randomised*  (women, n = …; infants, n = …) | | | | |  | | | |
|  | |  | | |  | | | | |  | |
|  | |  | | | | | | | |  | |
| Allocated to breastfeeding counselling and support and nutritional supplementation  (women, n = …; infants, n = …) | | | |  | | Allocated to breastfeeding counselling and support  (women, n = …; infants, n = …) | | | | | |
|  | |  | | | | | | |  | | |
| Lost to follow-up  (women, n = …; infants, n = …)  Withdrawn  (women, n = …; infants, n = …) |  |  | | | | | | |  | | Lost to follow-up  (women, n = …; infants, n = …)  Withdrawn  (women, n = …; infants, n = …) |
|  |  |  |  |  |  |  |  |  |  | |  |
|  | |  | | | | | | |  | | |
| Infants analyzed (n = …, %)  Infants excluded from analyses (n = …, %)**  Infants with growth concern (n = …, %)  Infants with growth faltering (n = …, %) | | | |  | | Infants analyzed (n = …, %)  Infants excluded from analyses (n = …, %)**  Infants with growth concern (n = …, %)  Infants with growth faltering (n = …, %) | | | | | |

* Women randomised, with infants from multiple pregnancies allocated to the same group

** Reasons for exclusion to be listed

## **Appendix 3: Participant characteristics**

| Characteristic | | | | Population | | Intervention | | | Comparison |  |
| --- | --- | --- | --- | --- | --- | --- | --- | --- | --- | --- |
| **At baseline** | | | | | | | | | |  |
| Number of mothers | | |  | | |  | |  | |  |
| Number of infants | | |  | | |  | |  | |  |
|  | Singleton infants  Infants from multiple births | |  | | |  | |  | |  |
| *Infant characteristics* | | |  | | |  | |  | |  |
| Sex | | |  | | |  | |  | |  |
|  | Female  Male  Ambiguous/Unknown | |  | | |  | |  | |  |
| Birth weight (kg): | | |  | | |  | |  | |  |
|  | Mean (SD) | |  | | |  | |  | |  |
|  | Less than 2.5  2.5 or greater | |  | | |  | |  | |  |
| Term AGA  Term SGA  Preterm AGA  Preterm SGA | | |  | | |  | |  | |  |
| *Maternal Characteristics* | | |  | | |  | |  | |  |
| Maternal age (years): | | |  | | |  | |  | |  |
|  | Less than 20  20-29  30 or more  Not known | |  | | |  | |  | |  |
| Number of living children: | | |  | | |  | |  | |  |
|  | 1  2  3+ | |  | | |  | |  | |  |
| Mother’s highest educational level: | | |  | | |  | |  | |  |
|  | None  Primary  Secondary  More than secondary  Not known | |  | | |  | |  | |  |
| *Household characteristics* | | |  | | |  | |  | |  |
| Wealth quintile: | | |  | | |  | |  | |  |
|  | 1 (Lowest)  2  3  4  5 (Highest) | |  | | |  | |  | |  |
| Characteristic | | | | Population | | | Intervention | | | Comparison |
| *Feeding practice* | | | |  | |  | |  | | |
| Timing of initiation of breastfeeding in days  Mean (SD)  Median (IQR) | | | |  | |  | |  | | |
| Duration of any breastfeeding in weeks  Mean (SD)  Median (IQR) | | | |  | |  | |  | | |
| Breastfeeding in past 24 hours^*^: At enrolment | | | |  | |  | |  | | |
|  | | Exclusive breastfeeding  Predominant breastfeeding^*^  Partial breasteeding^*^  No breastfeeding | |  | |  | |  | | |
| Breastfeeding in past 24 hours^*^: At 1 completed month | | | |  | |  | |  | | |
|  | | Exclusive breastfeeding  Predominant breastfeeding^**^  Partial breasteeding^*^  No breastfeeding | |  | |  | |  | | |
| Breastfeeding in past 24 hours^*^: At 3 completed months | | | |  | |  | |  | | |
|  | | Exclusive breastfeeding  Predominant breastfeeding^*^  Partial breasteeding^*^  No breastfeeding | |  | |  | |  | | |
| Breastfeeding in past 24 hours^*^: At 5 completed months | | | |  | |  | |  | | |
|  | | Exclusive breastfeeding  Predominant breastfeeding^*^  Partial breasteeding^*^  No breastfeeding | |  | |  | |  | | |
| Any non NMS formula use since the last visit^†^: | | | |  | |  | |  | | |
|  | | | At enrolment  At 1 completed month  At 3 completed months  At 5 completed months |  | |  | |  | | |

^*^ Data obtained by maternal recall for the time period “in the past 24 hours” unless otherwise stated

^**^ Predominant breastfeeding is defined as the infant’s predominant source of nourishment being breastmilk, with the infant also have receiving water and water-based drinks (sweetened and flavoured water, teas, infusions, etc), fruit juice, and ritual fluids. Partial breastfeeding is defined as the infant receiving breastmilk as well as other milk or cereal, or other food.

^†^ Data obtained by maternal recall for the time period “since the last visit”

|  | **All enrolled infants** | | | | | **Intervention arm** | | | | | **Comparison arm** | | | | |
| --- | --- | --- | --- | --- | --- | --- | --- | --- | --- | --- | --- | --- | --- | --- | --- |
|  | **Total** | **Excl** | **Pred** | **Part** | **None** | **Total** | **Excl** | **Pred** | **Part** | **None** | **Total** | **Excl** | **Pred** | **Part** | **None** |
| **Breastfeeding practice:**  At enrolment  At 1 completed month  At 2 completed months  At 3 completed months  At 4 completed months  At 5 completed months |  |  |  |  |  |  |  |  |  |  |  |  |  |  |  |
|  | **All infants without Growth Faltering (GF)** | | | | | **Non-GF infants in Intervention arm** | | | | | **Non-GF infants in Comparison arm** | | | | |
|  | **Total** | **Excl** | **Pred** | **Part** | **None** | **Total** | **Excl** | **Pred** | **Part** | **None** | **Total** | **Excl** | **Pred** | **Part** | **None** |
| **Breastfeeding practice:**  At enrolment  At 1 completed month  At 2 completed months  At 3 completed months  At 4 completed months  At 5 completed months |  |  |  |  |  |  |  |  |  |  |  |  |  |  |  |
|  | **All infants with Growth Faltering (GF)** | | | | | **GF infants in Intervention arm** | | | | | **GF infants in Comparison arm** | | | | |
|  | **Total** | **Excl** | **Pred** | **Part** | **None** | **Total** | **Excl** | **Pred** | **Part** | **None** | **Total** | **Excl** | **Pred** | **Part** | **None** |
| **Breastfeeding practice:**  At enrolment  At 1 completed month  At 2 completed months  At 3 completed months  At 4 completed months  At 5 completed months |  |  |  |  |  |  |  |  |  |  |  |  |  |  |  |
| **Breastfeeding practice:**  Before GF identified  After GF identified |  |  |  |  |  |  |  |  |  |  |  |  |  |  |  |
| **Practice after GF identified:**  1 month after  2 months after  3 months after  4 months after |  |  |  |  |  |  |  |  |  |  |  |  |  |  |  |

## **Appendix 4: Serious adverse events**

|  | **Population** | **Intervention**  **n=xxxx** | **Comparison**  **n=xxxx** | **Risk Ratio**  **(95% CI)** | **p-value** |
| --- | --- | --- | --- | --- | --- |
| Number of enrolled mother-infant dyads |  |  |  |  |  |
| Death due to any cause in the first six months of life |  |  |  |  |  |
| Death associated with vomiting or diarrhoea in the first six months of life |  |  |  |  |  |
| Hospitalisation for any cause in the first six months of life |  |  |  |  |  |
| Hospitalisation for vomiting or diarrhoea in the first six months of life |  |  |  |  |  |
| Death or hospitalisation for any cause in the first six months of life |  |  |  |  |  |
| Death or hospitalisation associated with vomiting or diarrhoea in the first six months of life |  |  |  |  |  |

## **Appendix 5: Analysis of primary outcome**

|  | | **Population** | **Intervention** | **Comparison** |
| --- | --- | --- | --- | --- |
| Number (%) of infants with complete data on outcomes | |  |  |  |
|  | Singleton infants  Infants from multiple births |  |  |  |
| Post-randomisation deaths to 6 months (%) | |  |  |  |
| Wasted infants at 6 months (%) | |  |  |  |
| Total infants with primary outcome (mortality or wasting) (%) | |  |  |  |
| Mortality or wasting per 1000 infants  Unadjusted risk ratio (95% CI)  P value  Adjusted risk ratio (95% CI)^*^  P value | |  |  |  |

^*^ Adjusting for clustering due to multiple births, and with all stratification variables as covariates

## **Appendix 6: Analyses of secondary outcomes**

**Anthropometric outcomes**

|  | | Intervention | Comparison |
| --- | --- | --- | --- |
| Number of mothers | |  |  |
| Number of infants | |  |  |
|  | Singleton infants  Infants from multiple births |  |  |
| Number of infants followed to 6 months or died (%) | |  |  |
| Infants with severe wasting at 6 months  Unadjusted risk ratio (95% CI)  P value  Adjusted risk ratio (95% CI)^*^  P value | |  |  |
| Infants underweight at 6 months  Unadjusted risk ratio (95% CI)  P value  Adjusted risk ratio (95% CI)^*^  P value | |  |  |
| Infants with stunting at 6 months  Unadjusted risk ratio (95% CI)  P value  Adjusted risk ratio (95% CI)^*^  P value | |  |  |
| Infants with concurrent wasting and stunting at 6 months  Unadjusted risk ratio (95% CI)  P value  Adjusted risk ratio (95% CI)^*^  P value | |  |  |

^*^ Adjusting for clustering due to multiple births, and with all stratification variables as covariates

**Morbidity outcomes**

|  | | Intervention | Comparison |
| --- | --- | --- | --- |
| Number of mothers | |  |  |
| Number of infants | |  |  |
|  | Singleton infants  Infants from multiple births |  |  |
| Number of infants followed to 6 months or died (%) | |  |  |
| Infants with at least one all-cause hospital admission  Unadjusted risk ratio (95% CI)  P value  Adjusted risk ratio (95% CI)^*^  P value | |  |  |
| Infants with at least one hospital admission for diarrhoea  Unadjusted risk ratio (95% CI)  P value  Adjusted risk ratio (95% CI)^*^  P value | |  |  |
| Infants with at least one hospital admission for pneumonia  Unadjusted risk ratio (95% CI)  P value  Adjusted risk ratio (95% CI)^*^  P value | |  |  |
| Infants with at least one hospital admission for other infections  Unadjusted risk ratio (95% CI)  P value  Adjusted risk ratio (95% CI)^*^  P value | |  |  |
| Infants with at least one hospital admission for other causes  Unadjusted risk ratio (95% CI)  P value  Adjusted risk ratio (95% CI)^*^  P value | |  |  |

^*^ Adjusting for clustering due to multiple births, and with all stratification variables as covariates

**Care-seeking outcome**

|  | | Intervention | Comparison |
| --- | --- | --- | --- |
| Number of mothers | |  |  |
| Number of infants | |  |  |
|  | Singleton infants  Infants from multiple births |  |  |
| Number of infants followed to 6 months or died (%) | |  |  |
| Infants with at least one primary caregiver report of any health care-seeking  Unadjusted risk ratio (95% CI)  P value  Adjusted risk ratio (95% CI)^*^  P value | |  |  |

^*^ Adjusting for clustering due to multiple births, and with all stratification variables as covariates

**Breastfeeding practice outcome**

|  | | Intervention | Comparison |
| --- | --- | --- | --- |
| Number of mothers | |  |  |
| Number of infants | |  |  |
|  | Singleton infants  Infants from multiple births |  |  |
| Number of infants followed to 5 completed months or died (%) | |  |  |
| Breastfeeding practice in the past 24 hours, at 5 completed months (breastfeeding versus no breastfeeding)  Unadjusted risk ratio (95% CI)  P value  Adjusted risk ratio (95% CI)^*^  P value | |  |  |

^*^ Adjusting for clustering due to multiple births, and with all stratification variables as covariates
