# Supplementary material for: Nutritional management of growth faltering in infants aged under six months in Asia and Africa: study protocol for a multicentre randomised trial (BRANCH, BReAstfeediNg Counselling and management of growtH)
Source: Trials. 2025 Nov 6;26:474. doi: 10.1186/s13063-025-09034-y (PMC12590774; doi:10.1186/s13063-025-09034-y)
Supplement: Supplementary file 5 — Additional file 5: Appendix 5. Criteria for selecting sites [file 13063_2025_9034_MOESM5_ESM.docx]

**Title - Nutritional management of growth faltering in infants aged under six months in Asia and Africa. Study protocol for an individually randomised trial (BRANCH, BReAstfeediNg Counselling and management of growtH)**

**Authors – WHO BRANCH study group**

**Version date – 1Aug2025**

**Appendix 9. Criteria for selecting sites**

The World Health Organization coordinating unit invited 41 research teams working in South Asia or Sub-Saharan Africa to submit a five-page expression of interest (EOI). The 41 teams were invited based on their experience and strengths in conducting infant feeding and growth research.

Evaluation criteria were developed in advance and were listed in the call for EOI: (i) relevance of the study to the country/province, (ii) suitability of the site including the prevalence of malnutrition and feasibility of implementing the study in the site, (iii) strengths of the research team in areas required for implementing the trial, and (iv) experience of the research team in implementing breastfeeding interventions, supplementary feeding, and measurement of growth.

A total of 31 EOIs were received and screened using the predefined criteria by WHO team and 14 were shortlisted. An external panel of four independent global experts then rated the 14 EOIs using the evaluation criteria. The WHO coordination team then made the proposed selection of seven teams/sites (three from South Asia, four from sub-Saharan Africa) based on reviewers’ ratings of EOIs, prevalence of wasting and underweight in infants less than 6 months, country and region (EOIs from different countries and different regions preferred), national leadership of the research team and the feasibility of supporting the proposed budget.
