# Supplementary material for: Nutritional management of growth faltering in infants aged under six months in Asia and Africa: study protocol for a multicentre randomised trial (BRANCH, BReAstfeediNg Counselling and management of growtH)
Source: Trials. 2025 Nov 6;26:474. doi: 10.1186/s13063-025-09034-y (PMC12590774; doi:10.1186/s13063-025-09034-y)
Supplement: Supplementary file 6 — Additional file 6: Appendix 6. Criteria for selecting personnel [file 13063_2025_9034_MOESM6_ESM.docx]

**Title - Nutritional management of growth faltering in infants aged under six months in Asia and Africa. Study protocol for an individually randomised trial (BRANCH, BReAstfeediNg Counselling and management of growtH)**

**Authors – WHO BRANCH study group**

**Version date – 1Aug2025**

**Appendix 7. Criteria for selecting personnel delivering the interventions**

| Community-based peer counsellors. This is a team of lay workers, female, resident in the community for several years, with at least 10 years of schooling; preferably mothers with personal successful breastfeeding experience and motivated to help other mothers breastfeed. Peer counsellors will be trained using the adapted WHO Infant and Young Child Feeding Combined Course which includes provision of mental health support; |
| --- |
| Lactation counsellors. This is a team of workers who are female with formal training in health, at least 12 years of schooling and with prior training and experience in providing breastfeeding support including competency in supporting breastfeeding in preterm and SGA infants and with clinical/field experience in managing a small team e.g. community health workers. Additional training on psychosocial issues is needed and training will be provided if the worker does not have these skills. Lactation counsellors will be trained in the WHO 40-hour Breastfeeding Counselling Course. |
| Lactation consultants. This is a team of female senior health workers (may be a physician/senior nurse or midwife) who have received breastfeeding counselling training and has significant clinical/field experience including support of breastfeeding of preterm and SGA infants; also has proven experience in supervision of junior level staff. She will have had prior training in the WHO 40-hour Breastfeeding Counselling Course and will receive additional Trainer of Trainer support. The term lactation consultant in the study is used to denote a higher level of skill and responsibilities. The training and position are not equivalent to that of the International Board of Lactation Consultant Examiners (IBLCE) certified lactation consultant. |
| Clinical team – This is a team of qualified nurses and doctors who have received training in World Health Organization (WHO) clinical management which includes Integrated Management of Childhood Illnesses (IMCI) and the WHO Guidelines For Management Of Common Childhood Illnesses. |
| Supplementation team This is a team of qualified nurses and doctors who have received training in World Health Organization (WHO) clinical management which includes Integrated Management of Childhood Illnesses (IMCI) and the WHO Guidelines For Management Of Common Childhood Illnesses. |
| Surveillance team. Trained research staff who have completed at least secondary school education. |
