# Supplementary figures and images for: Nutritional management of growth faltering in infants aged under six months in Asia and Africa: study protocol for a multicentre randomised trial (BRANCH, BReAstfeediNg Counselling and management of growtH)
Source: Trials. 2025 Nov 6;26:474. doi: 10.1186/s13063-025-09034-y (PMC12590774; doi:10.1186/s13063-025-09034-y)

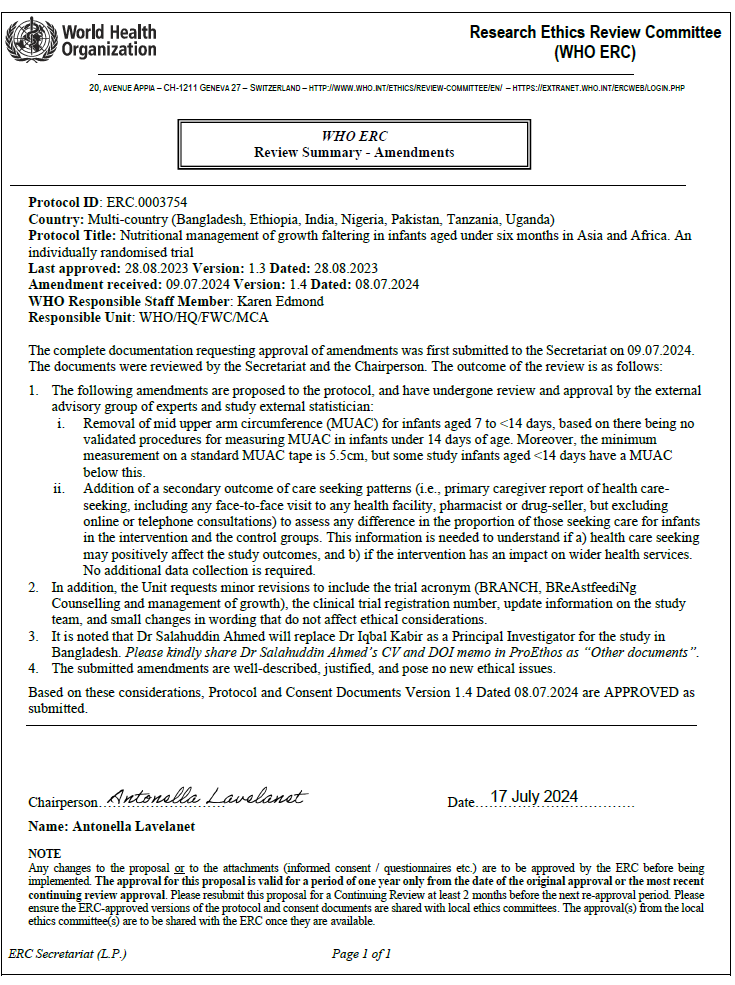

Supplement: Supplementary file 7 — Additional file 7: Appendix 7. WHO ethical approval [file 13063_2025_9034_MOESM7_ESM.docx]

**Appendix 8 BRANCH funding proof Gates Foundation committed grants INV 025356**


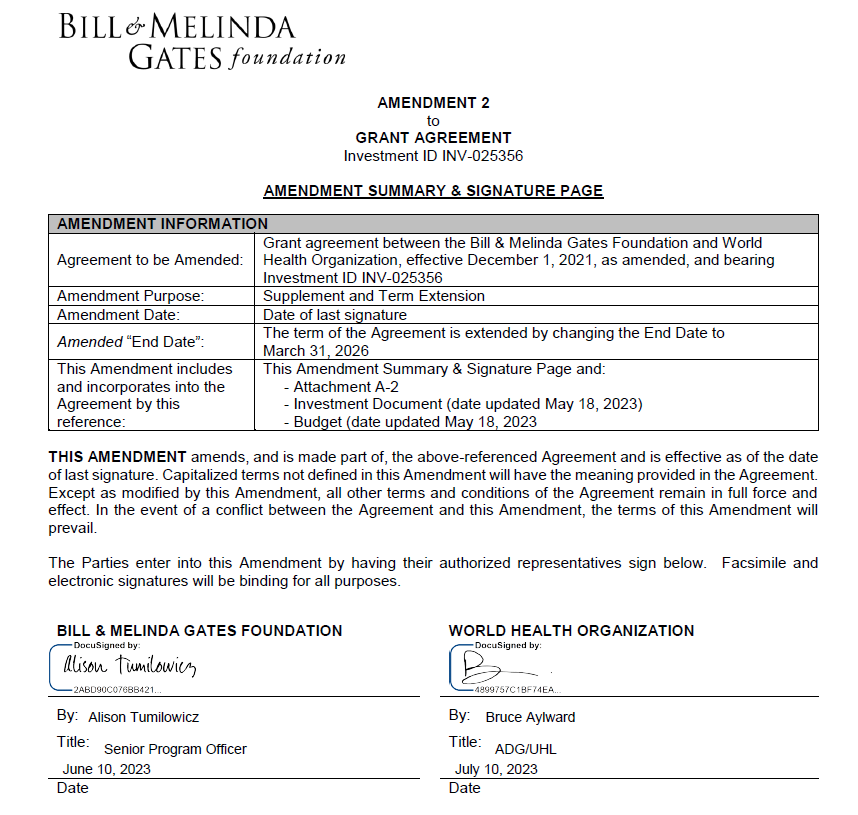


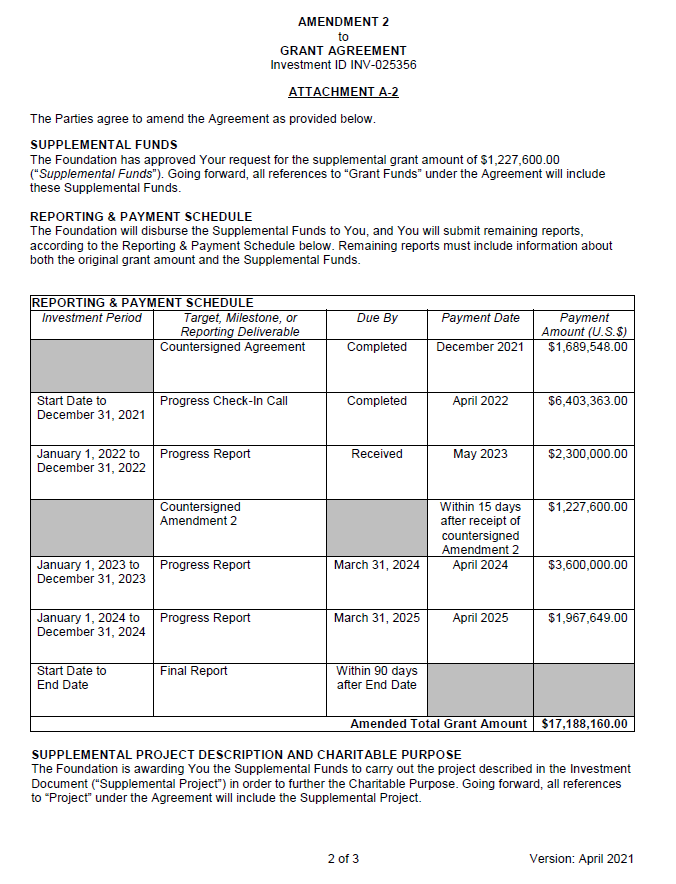


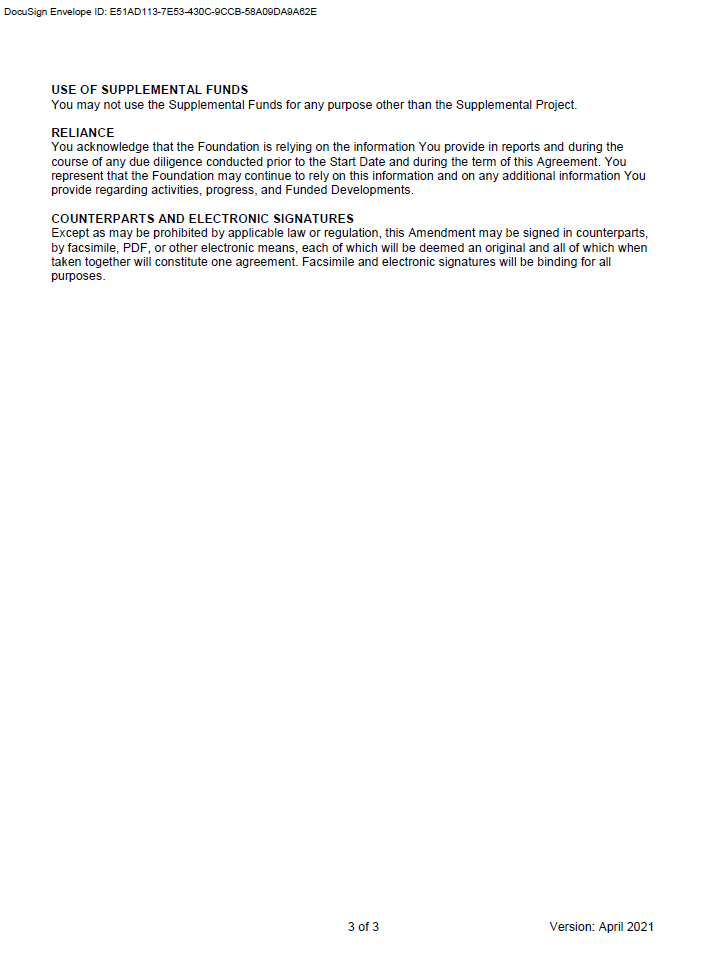

Supplement: Supplementary file 8 — Additional file 8: Appendix 8. Funding information [file 13063_2025_9034_MOESM8_ESM.docx]
